# Novel 7-Deazapurine Incorporating Isatin Hybrid Compounds as Protein Kinase Inhibitors: Design, Synthesis, In Silico Studies, and Antiproliferative Evaluation

**DOI:** 10.3390/molecules28155869

**Published:** 2023-08-04

**Authors:** Mohammed M. Alanazi, Ashwag S. Alanazi

**Affiliations:** 1 Department of Pharmaceutical Chemistry, College of Pharmacy, King Saud University, P.O. Box 2457, Riyadh 11451, Saudi Arabia; 2 Department of Pharmaceutical Sciences, College of Pharmacy, Princess Nourah Bint Abdulrahman University, Riyadh 11671, Saudi Arabia

**Keywords:** anticancer, protein kinase inhibitor, 7-deazapurine, isatin

## Abstract

Cancer is a multifactorial disorder with extremely complex genetics and progression. The major challenge in cancer therapy is the development of cancer resistance and relapse. Conventional anticancer drugs directly target the DNA of the cell, while modern chemotherapeutic drugs include molecular-targeted therapy, such as targeting the abnormal cell signaling inside the cancer cells. Targeted chemotherapy is effective in several malignancies; however, the success has always been limited by drug resistance and/or side effects. Anticancer with multi-targeted actions simultaneously modulates multiple cancer cell signaling pathways and, therefore, may ease the chance of effective anticancer drug development. In this research, a series of 7-deazapurine incorporating isatin hybrid compounds was designed and successfully synthesized. Among those hybrids, compound **5** demonstrated a very potent cytotoxic effect compared to the reference anticancer drug against four cancer cell lines. Likewise, compound **5** inhibited the activity of four protein kinase enzymes in nanomolar ranges. Further analysis of the biological evaluation of compound **5** revealed the capability of compound **5** to arrest cell cycle progression and induce programmed cell death. Moreover, molecular simulation studies were performed to investigate the possible types of interactions between compound **5** and the investigated protein kinases. Finally, taking into consideration all the abovementioned findings, compound **5** could be a good candidate for further investigations.

## 1. Introduction

Kinases are enzymes that catalyze the phosphorylation of biomolecules that control complex cellular processes, such as growth, proliferation, differentiation, motility, and apoptosis [1,2]. In some cases, kinase enzymes become overactive and consequently lead to cancer. Since 1999, more than 80 kinase inhibitors have been approved, mostly to treat cancer [3]. The success of this class of drugs in cancer treatment makes kinase enzymes a target in anticancer drug discovery research [4].

Kinase inhibitors are classified according to the binding site to type I, I1/2, II, III, and IV [5,6]. Kinase inhibitors type I bind in the active kinase ATP-binding pocket, while types I1/2 and II bind with the hinge region, connecting the small and large lobes of the enzyme [3,5]. Types III and IV are allosteric inhibitors (non-ATP-competitive), with type III being bound next to the ATP-binding site and type IV away from the ATP-binding site [3].

Targeted therapy is one of the most promising methods for cancer therapy because it may benefit in improving efficacy and reducing toxicity. Multikinase inhibitors, especially tyrosine kinase inhibitors targeting specific tyrosine kinases within the tumor cell, are a very promising class of targeted anticancer agents [7]. Osimertinib and cabozantinib are multikinase inhibitors used for the treatment of lung and kidney cancer, respectively, and they promptly secured a role in standard treatment for new indications [8].

Protein tyrosine kinases (PTKs) are enzymes that phosphorylate amino acid residues in proteins, lead to proteins’ 3D structural change, and ultimately affect protein function [9]. In cancer, more than 60% of oncoproteins and proto-oncoproteins are produced by PTKs [9]. Several cancers have revealed overactivation of vascular endothelial growth factor receptor2 (VEGFR2), human epidermal receptor (HER2), and endothelial growth factor receptors (EGFRs). Activation of VEGFR2 by vascular endothelial growth factor (VEGF) initiates the process of phosphorylation, which increases the proliferation and migration of endothelial cells; therefore, blocking this signaling pathway will lead to the suppression of tumor growth [10,11,12]. Moreover, poor prognoses of several human cancers is associated with the overexpression of HER2 [13]. The overexpression of HER2 was found to stimulate EGFR signaling. Therefore, the inhibition of the EGFR and HER2 pathways is of great clinical interest [14].

On the other hand, cyclin-dependent kinases (CDKs) are serine–threonine protein kinases that control cell growth and division [15]. It was determined that inhibiting the action of CDKs controls the cellular overgrowth found in several sorts of cancers. One of the fundamental CDKs associated with inadequate cancer progression is the overexpression of CDK2. Thus, controlling the overexpression of CDK2 could lead to a reversed malignancy phenotype in cancer cells [16]. Therefore, inhibition of EGFR, HER2, VEGFR2, and CDK2 proteins is an outstanding medicinal target for the development of new anticancer drugs [17]. Following this strategy (mutikinase inhibition) in cancer treatment will avoid treating cancer with either single drug treatment, which is unable to destroy all cells holding multiple stimuli, or using multiple anticancer agents that will increase toxicity and drug–drug interaction [18].

**Figure 1:**
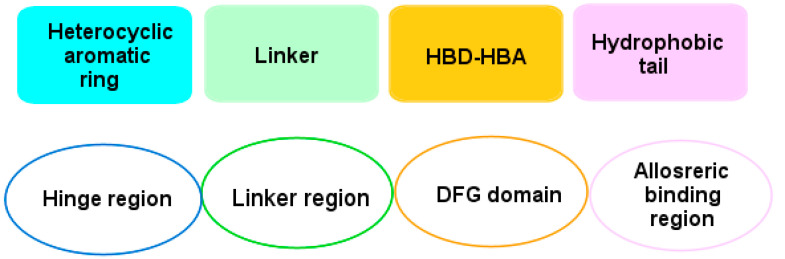
Important pharmacophoric features of protein kinase inhibitors and different regions in the ATP-binding site. Hing region, linker region, DFG domain, and allosteric-binding region are the main binding sites in the target kinase enzymes.

Previously, we reported some derivatives of 7-deazapurin linked to substituted isatins through hydrazine as a linker [19]. However, we established that the hydrazine linker does not have the ability to bind strongly to the linker region and the DFG region in the target kinase enzymes. In fact, the basic pharmacophoric features of protein kinase inhibitors such as VEGFR-2 inhibitors are a pharmacophore moiety that binds to the hinge region, a spacer that binds to the DFG domain and the linker region, and a flat heteroaromatic ring that binds to the allosteric-binding region (Figure 1) [20,21,22,23]. In this study, we designed new compounds in continuation to our previous work [19], where 7-deazapurin was linked with different isatins with a short linker (hydrazine). We hypothesize that linking the two motilities with a long linker (4-aminobenzohydrazide) will allow for better interactions with the DFG domain and the linker region, which may improve the kinase inhibition activity. These new compounds are designed as anticancer agents with multi-target effects.

## 2. Results and Discussion

### 2.1. Design

**Figure 2:**
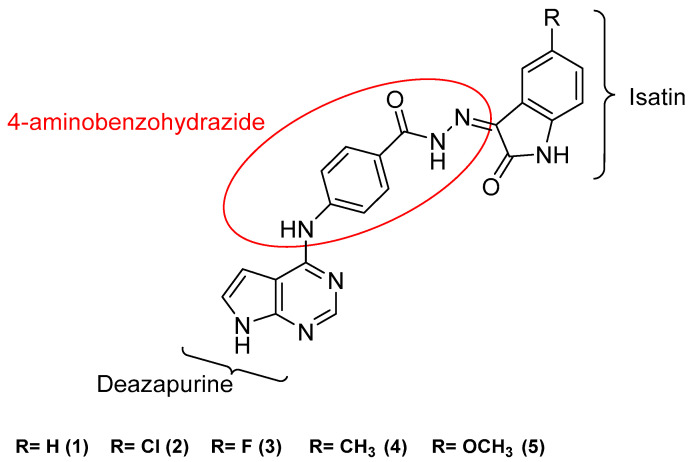
Design of isatin–deazapurine hybrid compounds **1**–**5** comprising three parts. 7-deazapurin and substituted isatins are proposed to bind hinge region and the allosteric-binding site while the 4-aminobenzohydrazide is proposed to bind the linker and DFG regions in the target kinase enzymes.

The aim of this study is to design and synthesize novel isatin–deazapurine hybrid compounds linked with 4-aminobenzohydrazide (compounds **1**–**5**) (Figure 2). First, the physicochemical properties, pharmacokinetic, and toxicity profiles of the target compounds **1**–**5** were predicted in silico using pkCSM website (Table 1) [24]. The physicochemical properties of designed compounds **1**–**5** were calculated by following Lipinski’s rule of 5, which is one strategy used as guidelines for what may constitute a successful drug. The pharmacokinetic parameters, absorption, distribution, metabolism, excretion, and toxicity (ADMET), were also predicted by pkCSM. These in silico calculations were followed to ensure that the target compounds **1**–**5** can reach the kinase enzymes and produce their physiological effect.

According to the results (Table 1), all compounds followed Lipinski’s rule of five, where all molecular weights were less than 500, log p less than 5, and hydrogen bond donor and acceptor less than 5 and 10, respectively, for all compounds. In comparison to sunitinib, all compounds showed comparable results in terms of absorption, distribution, excretion, and toxicity. These results indicate that all designed compounds may have good oral bioavailability and safety profiles. However, all designed compounds are predicted to inhibit CYP enzymes, such as 1A2, 2C19, 2C9, and 3A4. 

### 2.2. Chemistry

In a continuation of our previous study [17], we report here the design and synthesis of novel 7-deazapurine incorporating isatin hybrid compounds. In this work, isatin was linked with 7-deazapurine to enhance lipophilicity. 

**Scheme 1:**
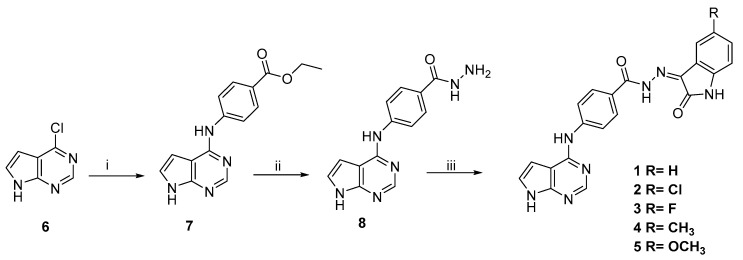
Design of isatin–deazapurine hybrid compounds **1**–**5**. Reagents and conditions: i. Ethyl 4-aminobenzoate, abs. EtOH, reflux, 7 h. ii. N_2_H_2_. H_2_O, abs. EtOH, reflux, 5 h. iii. Appropriate isatin, AcOH, EtOH, reflux, 6–9 h.

Initially, the ethyl 4-aminobenzoate **6** was added to a solution of 4-chloro-7H-pyrrolo[2,3-d]pyrimidine in absolute ethanol and stirred at reflux for 7 h. The solid obtained **7** was filtered and washed with cold water with a yield of 91.13% and used for the next step. Then, ethyl 4-((7H-pyrrolo[2,3-d]pyrimidin-4-yl)amino)benzoate **7** was reflexed in excess of hydrazine hydrate for 5 h. The reaction mixture was cooled using ice water to give resultant precipitate **8**, which was filtered, washed thoroughly with water, and dried for the next step, with a yield of 76.92%. Finally, the intermediate **9**, appropriate isatin, and glacial acetic acid were mixed in absolute ethanol and refluxed for 6–9 h. The reaction mixture was cooled, and the formed precipitate was filtered, washed with water, and recrystallized to obtain the final compounds **1**–**5**, with a yield of 88.85–94.41% (Scheme 1). The intermediates **7** and **8** and the final compounds **1**–**5** were fully characterized by ^1^H, ^13^C NMR, and LCMS. High-performance liquid chromatography (HPLC) analysis demonstrated purity > 95% (Supplementary Materials).

### 2.3. Biological Evaluation

#### 2.3.1. In Vitro Cytotoxic Assay against HepG2, MCF-7, MDA-MB-231, and HeLa Cell Lines

Biological investigation of the synthesized derivatives **1**–**5** involved the cytotoxic potential of these new derivatives against human carcinoma cell lines in comparison to doxorubicin. Cytotoxicity of the synthesized compounds was measured using MTT assay against hepatocellular carcinoma (HepG2), mammary gland cancer (MCF-7), breast cancer (MDA-MB-231), and epithelioid cervix carcinoma (HeLa) (Table 2). The results indicate that compound **5** has a potent antiproliferative activity with IC_50_ values of 6.11 ± 0.4, 5.93 ± 0.3, 2.48 ± 0.1, and 1.98 ± 0.1 µM against HepG2, MCF-7, MDA-MB-231, and HeLa cell lines, respectively. Compound **1** revealed moderate antiproliferative activity against all tested cell lines, with other compounds demonstrating weak activity. In fact, compound **5** with a methoxy substitution was the most potent derivative over all cancer cell lines, and, therefore, it was selected for further investigations, including kinase inhibition assays and apoptosis. 

#### 2.3.2. Kinase Inhibition Assay over CDK2, EGFR, HER2, and VEGFR2

Compound **5** was assessed for inhibitory activity against CDK2, EGFR, HER2, and VEGFR2 enzymes (Table 3). The results were obtained as the 50% kinase inhibition concentration values in comparison to reference drugs. Interestingly, the IC_50_ value of compound **5** (0.081 ± 0.002 μM) revealed promising HER2 inhibitory activity. In addition, compound **5** showed comparable results as the reference drugs, ribocicli, erlotinib, lapatinib, and sorafenib, over all the tested proteins. Actually, compound **5** exhibited multikinase inhibition and, accordingly, compound **5** was further evaluated for apoptosis and cell cycle analysis.

#### 2.3.3. Cell Cycle Analysis

**Figure 3:**
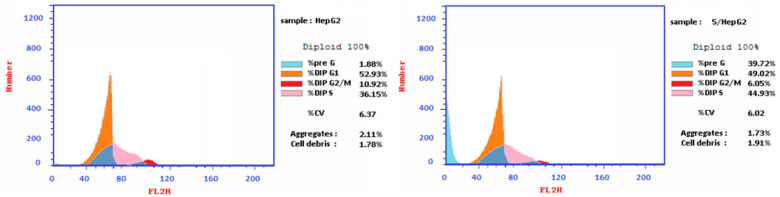
Cell cycle analysis of HepG2 cells treated with compound **5**. HepG2 cells accumulation in different phases of cell cycle after 24 h incubation with compound **5** or no treatment.

Cell cycle analysis can determine at which phase in the cell cycle apoptosis occurs. The cell cycle is four phases consisting of G1 (growth), S (DNA synthesis), G2 (growth and preparation for mitosis), and M (mitosis). Normal cell growth and proliferation are controlled by various checkpoints at these phases. Thus, HepG2 cells were treated with 6.11 μM of compound **5** to determine at which phase apoptosis occurs in the cell cycle for 24 h (Figure 3 and Table 4). As a result, compound **5** arrested the cell cycle of HepG2 cells at G1 and S phases, which confirms the antiproliferative effect of compound **5**.

#### 2.3.4. Apoptosis Analysis

**Figure 4:**
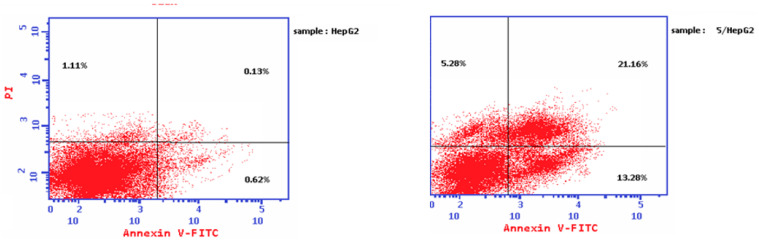
Flow cytometry analysis of apoptosis in HepG2 cells treated with compound **5**. HepG2 were treated with compound **5** or with no treatment and Dual-stained with Annexin V-FITC and PI for detection of apoptotic cells.

Annexin V and propidium iodide (PI) reagent were used to detect the cell death in apoptosis by binding to phosphatidyl serine and DNA in plasma membrane, respectively. The cell death was examined by staining HepG2 cells with Annexin V and PI, and cellular fluorescence analysis was then performed using flow cytometry (Table 5). According to the results, compound **5** induced apoptosis by 160-fold in the late stage and by 20-fold in the early stage. Furthermore, treatment with HepG2 demonstrated an increase in necrotic cell death (4.75 fold) compared to untreated cells. As shown in the dot plot results, compound **5** caused exerted noticeable cell damage compared to untreated cells (Figure 4). Collectively, the results of cell cycle and apoptosis analysis confirmed the cytotoxicity effects of compound **5**.

#### 2.3.5. Caspase 3, Caspase 9, BAX, and Bcl-2 Level Protein Assays

The effect of compound **5** was also evaluated on caspase 3, caspase 9, BAX, and Bcl-2 protein levels in HepG2 (Table 6). The results showed that compound **5** caused an 8-fold, 5-fold, and 6-fold increase in caspase 3, caspase 9, and BAX gene expression, respectively. Additionally, the level of Bcl-2 was reduced in the treated cells (0.18-fold) compared to untreated control cells. 

### 2.4. Molecular Docking

Molecular docking analysis was studied to predict the potential binding interactions between compound **5** and the tested protein kinases. Hence, compound **5** was docked into the active binding sites of EGFR, VEGFR2, HER2, and CDK2 using 4hjo, 4asd, 3rcd, and 3ti1 X-ray crystal structures, respectively. The obtained results were analyzed based on the comparison with the co-crystallized ligands, erlotinib, sorafenib, TAK-285, and sunitinib for the abovementioned proteins, respectively. At the beginning of the docking experiments, the co-crystallized ligands were redocked into the respective proteins to validate the docking process. The results showed that all the redocked ligands had the same alignment with the native co-crystallized poses. 

**Figure 5:**
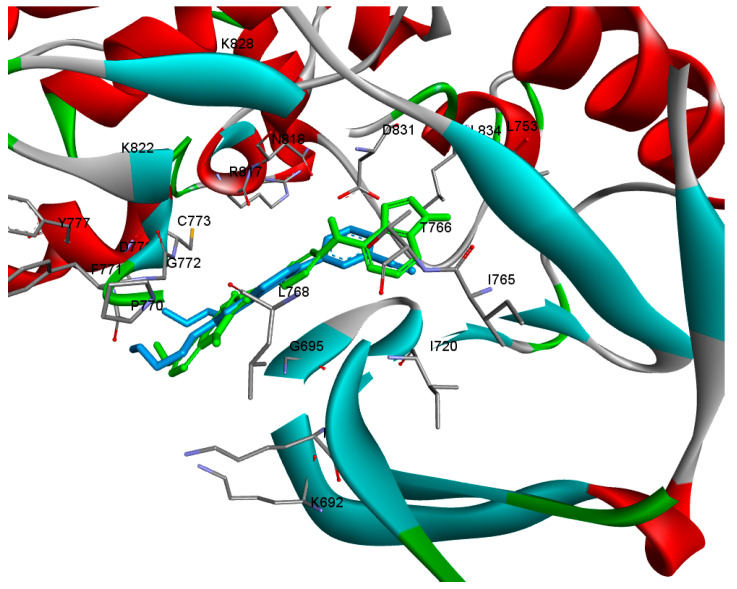
Alignment of compound 5 (green) with erlotinib (blue) in the active site of EGFR.

**Figure 6:**
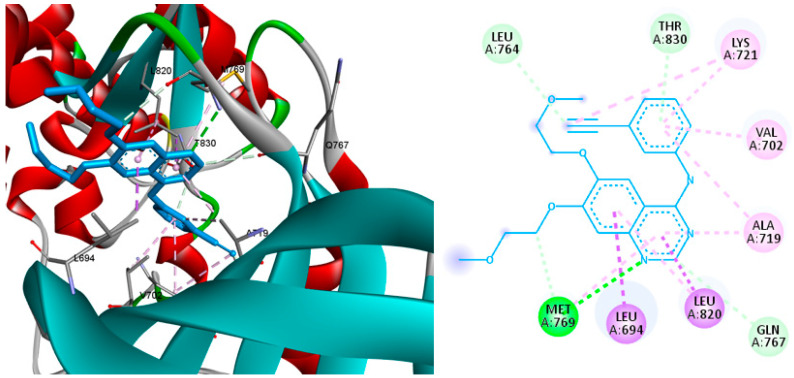
Schematic of 3D and 2D interactions of erlotinib with the active site of EGFR. Green dotted lines represent hydrogen bonds.

**Figure 7:**
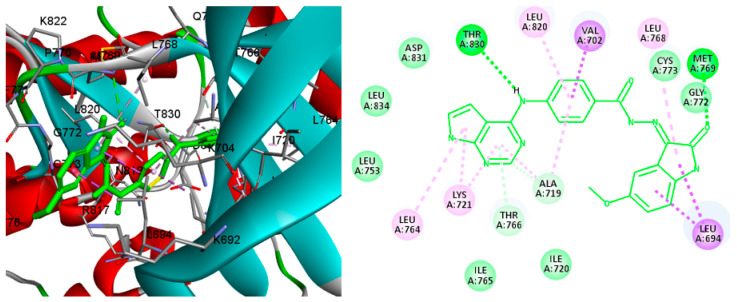
Schematic of 3D and 2D interactions of compound **5** with the active site of EGFR. Green dotted lines represent hydrogen bonds.

First, docking compound **5** into the active site of EGFR revealed that compound **5** superimposed erlotinib and stabilized by making two hydrogen bonds with Thr830 and Met769, while erlotinib made only one hydrogen bond with Met769. On the other hand, both compound **5** and erlotinib had almost the same hydrophobic interactions with Lys721, Leu826, Val702, Ala719, Leu768, and Leu694 (Figure 5, Figure 6 and Figure 7).

**Figure 8:**
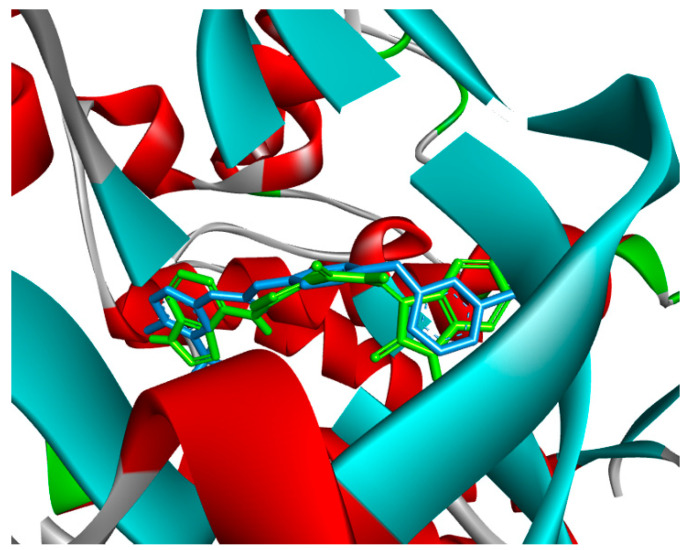
Alignment of compound 5 (green) with sorafenib (blue) in the active site of VEGFR2.

**Figure 9:**
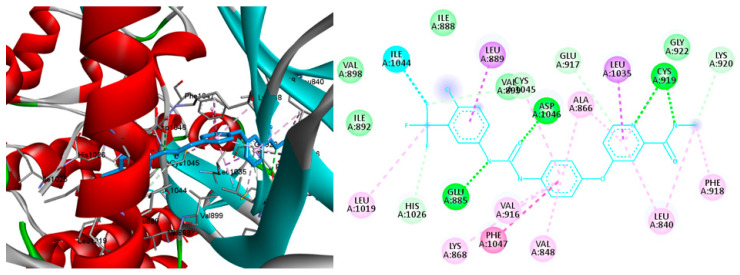
Schematic of 3D and 2D interactions of sorafenib in the active site of VEGFR2. Green dotted lines represent hydrogen bonds.

**Figure 10:**
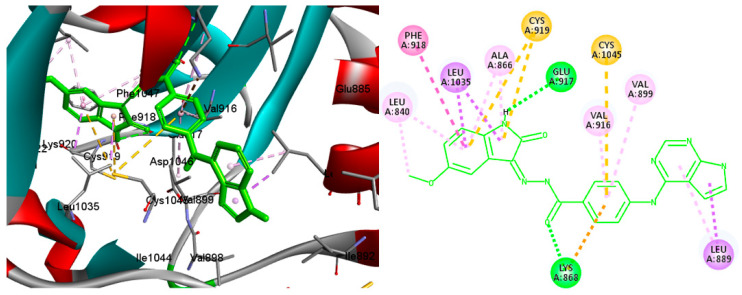
Schematic of 3D and 2D interactions of compound **5** in the active site of VEGFR2. Green dotted lines represent hydrogen bonds.

Second, following the docking of compound **5** and sorafenib into the active site of VEGFR2, the results revealed the superimposition of the two compounds in the active site (Figure 8). Unlike sorafenib, which is stabilized by making four hydrogen bonds with Glu885, Asp 1046, and Cys919 (two), compound **5** made only two hydrogen bonds with glu917 and lys868. Likewise, sorafenib performed more nonhydrogen bonding interactions with Ile1044, Leu1019, His1026, Lys868, Phe1047, Val848, Lys920, and Ala866, while the two compounds interacted equally with Val916, Leu840, Phe918, Leu1035, Cys1045, and Leu889 (Figure 9 and Figure 10).

**Figure 11:**
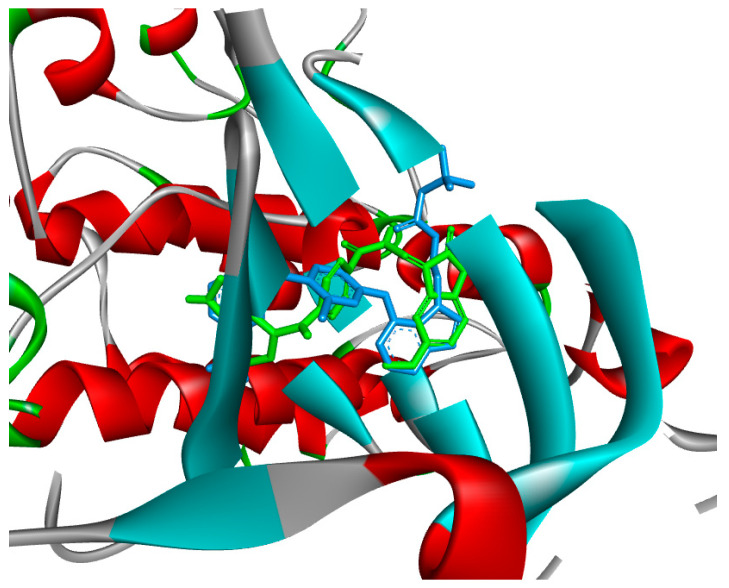
Alignment of compound **5** (green) with lapatinib (blue) in the active site of HER2.

**Figure 12:**
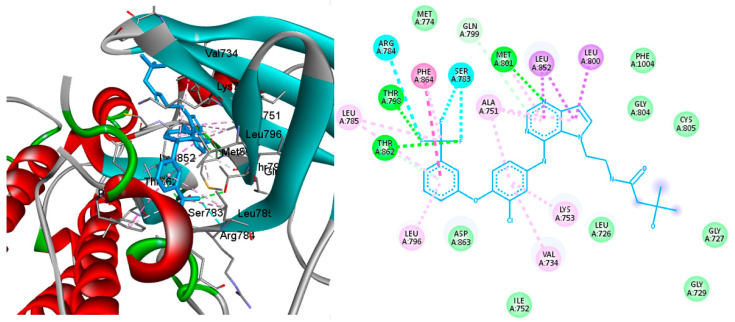
Schematic of 3D and 2D interactions of lapatinib in the active site of HER2. Green dotted lines represent hydrogen bonds.

**Figure 13:**
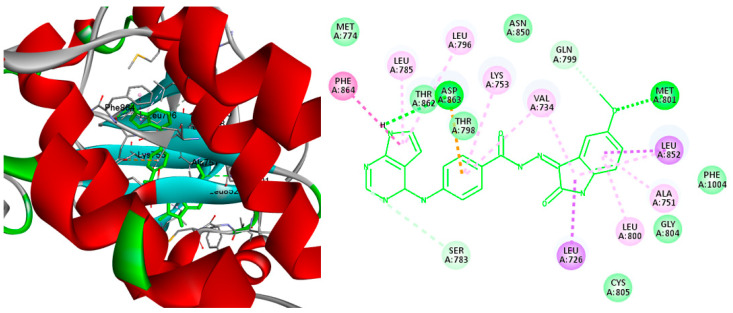
Schematic of 3D and 2D interactions of compound **5** in the active site of HER2. Green dotted lines represent hydrogen bonds.

Third, docking lapatinib and compound **5** into the active site of HER2 enzyme revealed the superimposition of the two compounds in the ATP active binding of the kinase enzyme (Figure 11). Lapatinib interacted with Thr798, Thr862, and Met801 via hydrogen bonding, while compound **5** interacted only with Thr862 and Met801. The two compounds stabilized through similar hydrophobic interactions, except for lapatinib, which made two extra interactions with Ag784 and Lys751 (Figure 12 and Figure 13).

**Figure 14:**
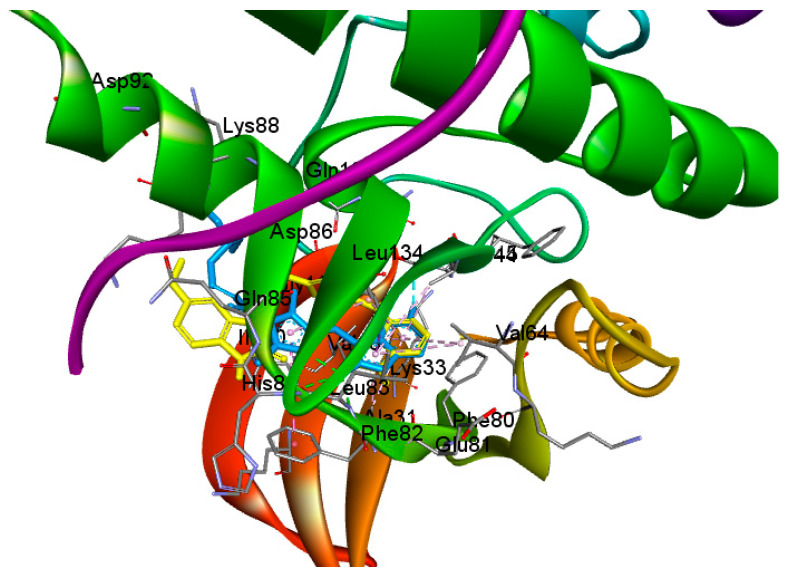
Alignment of compound **5** (yellow) with sunitinib (blue) in the active site of CDK2.

**Figure 15:**
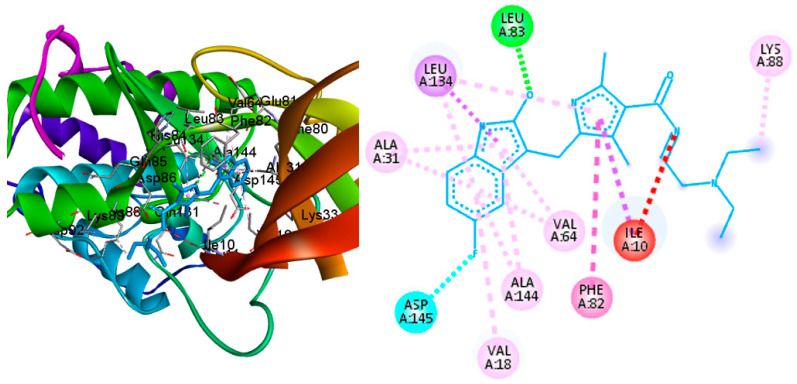
Schematic of 3D and 2D interactions of sunitinib in the active site of CDK2. Green dotted lines represent hydrogen bonds.

**Figure 16:**
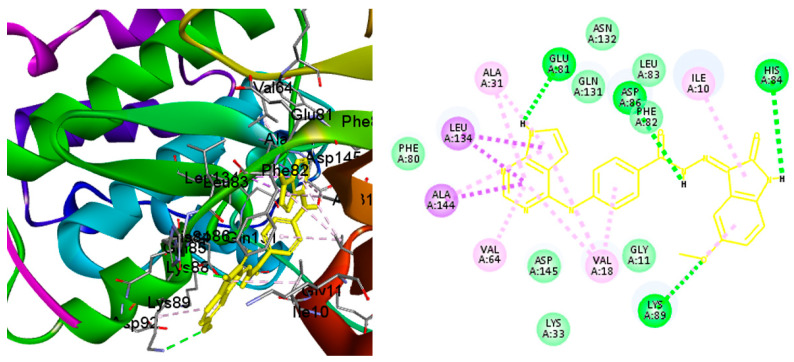
Schematic of 3D and 2D interactions of compound **5** in the active site of CDK2. Green dotted lines represent hydrogen bonds.

Fourth, compound **5** and sunitinib were docked into the active site of CDK2. Figure 14 depicts the alignment of the two compounds in the ATP-binding site of the CDK2 enzyme. It is notable in Figure 15 and Figure 16 that both compounds made similar types of hydrophobic interactions. Compound **5** was stabilized by three hydrogen bonds with Glu81, Lys89, and His84, as compared to sunitinib, which was only stabilized by one hydrogen bond with Leu88.

Finally, to compare the binding interactions of compound **5** and other synthesized compounds, compounds **1**–**4** were docked into the active site of CDK2. It seems that the hydrogen bond made between the Lys89 and the oxygen atom of the methoxy group in compound **5** is a key interaction for a strong binding with the enzyme, which could not happen with the other compounds due to the lack of a hydrogen bond making substitution at the same position (Supplementary Materials).

## 3. Material and Methods

### 3.1. Chemistry

#### 3.1.1. Preparation of Compounds **1**–**5**

A mixture of 4-((7H-pyrrolo[2,3-d]pyrimidin-4-yl)amino)benzohydrazide (0.2 gm, 0.7 mmol), appropriate isatin (0.7 mmol), and glacial acetic acid (1 mL) in absolute ethanol (20 mL) was refluxed for about 6–9 h. The reaction mixture was cooled and, after adding ice water, the formed precipitate was filtered off, washed with water, and recrystallized from the proper solvent to obtain the desired compound. 

*4-((7H-pyrrolo[2,3-d]pyrimidin-4-yl)amino)-N′-(2-oxoindolin-3-ylidene)benzohydrazide* (**1**), Yellow solid, Yield 88.85%; m.p. > 300 °C; ^1^H NMR (700 MHz, DMSO-*d*_6_): δ 13.97 (s, 1H), 13.29 (s, 1H), 11.48 (s, 1H), 9.74 (s, 1H), 8.32 (s, 1H), 8.20 (d, 2H), 7.97 (d, 2H), 7.59 (d, 1H), 7.39 (m, 2H), 7.24–6.73 (m, 3H). ^13^C NMR (176 MHz, DMSO) δ 163.62 (C), 162.95 (C), 153.34 (C), 151.68 (C), 150.97 (CH), 145.46 (C), 142.76 (C), 137.94 (C), 132.06 (CH), 128.85 (CH), 124.83 (CH), 123.57 (CH), 123.21 (CH), 121.32 (C), 120.45 (CH), 119.71 (CH), 111.68 (2xCH), 104.83 (C), 99.22 (CH). LC-MS *m*/*z* 395.9 [M−H]^−^, 398.2 [M+H]^+^. HPLC (reversed-phase) 0.1% formic acid/Acetonitrile 90:10 to 10:90 in 30 min, λ = 4,767,400 mAU, tR = 19.619 min (99%). Anal. Calcd. for C_21_H_15_N_7_O_2_ (397.12): C, 63.47; H, 3.8; N, 24.67; found C, 63.39; H, 3.84; N, 24.70%.

*4-((7H-pyrrolo[2,3-d]pyrimidin-4-yl)amino)-N′-(5-chloro-2-oxoindolin-3-ylidene)benzohydrazide* (**2**), Yellow solid, Yield 94.41%; m.p. > 300 °C; ^1^H NMR (700 MHz, DMSO) δ 13.92 (s, 1H), 11.91 (s, 1H), 11.51 (s, 1H), 9.74 (s, 1H), 8.41 (s, 1H), 8.16 (s, 2H), 8.10–7.83 (s, 2H), 7.62 (s, 1H), 7.45 (s, 1H), 7.27 (s, 1H), 7.12–6.91 (s, 1H), 6.89 (s, 1H). ^13^C NMR (176 MHz, DMSO) δ 163.44 (C), 153.33 (C), 151.67 (C), 150.94 (C), 145.59 (CH), 141.42 (C), 136.97 (C), 131.44 (C), 128.98 (2xCH), 127.34 (CH), 124.58 (C), 123.50 (CH), 122.28 (C), 120.82 (CH), 119.69 (C), 113.30 (2xCH), 104.85 (C), 99.20 (CH). LC-MS *m*/*z* 429.9 [M−H]^−^, 431.9 [M+H]^+^. HPLC (reversed-phase) 0.1% formic acid/Acetonitrile 90:10 to 10:90 in 30 min, λ = 961,476 mAU, tR = 22.824 min (96%). Anal. Calcd. for C_21_H_14_ClN_7_O_2_ (431.09): C, 58.41; H, 3.27; N, 22.7; found C, 58.56; H, 3.22; N, 22.88%.

*4-((7H-pyrrolo[2,3-d]pyrimidin-4-yl)amino)-N′-(5-fluoro-2-oxoindolin-3-ylidene)benzohydrazide* (**3**), Yellow solid, Yield 90.94%; m.p. > 300 °C; ^1^H NMR (700 MHz, DMSO) δ 13.98 (s, 1H), 11.87 (s, 1H), 11.41 (s, 1H), 9.54 (s, 1H), 8.57 (s, 1H), 8.28–8.10 (m, 2H), 8.10–7.81 (m, 2H), 7.45 (s, 1H), 7.41–7.20 (m, 2H), 7.12–6.77 (m, 2H). ^13^C NMR (176 MHz, DMSO) δ 163.75 (C), 159.57 (C), 158.21 (C), 153.33 (C), 151.67 (CH), 150.99 (C), 145.58 (C), 139.00 (C), 128.94 (2xCH), 124.62 (CH), 123.50 (C), 121.72 (C), 119.69 (2xCH), 118.39 (CH), 112.79 (CH), 108.42 (CH), 104.84 (C), 99.20 (CH). LC-MS *m*/*z* 413.9 [M−H]^−^, 414.9 [M+H]^+^. HPLC (reversed-phase) 0.1% formic acid/Acetonitrile 90:10 to 10:90 in 30 min, λ = 5,218,874 mAU, tR = 20.708 min (99%). Anal. Calcd. for C_21_H_14_FN_7_O_2_ (415.12): C, 60.72; H, 3.4; N, 23.6; found C, 60.59; H, 3.32; N, 23.42%

*4-((7H-pyrrolo[2,3-d]pyrimidin-4-yl)amino)-N′-(5-methyl-2-oxoindolin-3-ylidene)benzohydrazide* (**4**), Yellow solid, Yield 93.37%; m.p. > 300 °C; ^1^H NMR (700 MHz, DMSO) δ 13.97 (s, 1H), 11.87 (s, 1H), 11.39 (s, 1H), 9.48 (s, 1H), 8.38 (s, 1H), 8.19 (m, 2H), 8.07–7.72 (m, 2H), 7.64–6.98 (m, 3H), 6.98–6.51 (m, 2H), 2.34 (s, 3H). ^13^C NMR (176 MHz, DMSO) δ 163.68 (C), 153.35 (C), 151.66 (C), 150.99 (CH), 145.43 (C), 140.50 (C), 132.48 (C), 132.30 (CH), 128.83 (2xCH), 124.84 (C), 123.46 (C), 121.68 (CH), 120.44 (CH), 119.46 (C), 111.45 (2xCH), 104.82 (C), 99.20 (CH), 21.02 (CH3). LC-MS *m*/*z* 409.9 [M−H]^−^, 410.9 [M+H]^+^. HPLC (reversed-phase) 0.1% formic acid/Acetonitrile 90:10 to 10:90 in 30 min, λ = 66,504 mAU, tR = 15.149 min (95%). Anal. Calcd. for C_22_H_17_N_7_O_2_ (411.14): C, 64.23; H, 4.17; N, 23.83; found C, 64.32; H, 4.18; N, 23.98%.

*4-((7H-pyrrolo[2,3-d]pyrimidin-4-yl)amino)-N′-(5-methoxy-2-oxoindolin-3-ylidene)benzohydrazide* (**5**), Brown solid, Yield 92.26%; m.p. > 300 °C; ^1^H NMR (700 MHz, DMSO) δ 14.04 (s, 1H), 11.80 (s, 1H), 11.21 (s, 1H), 9.73 (s, 1H), 8.30 (m, 1H), 8.34 (m, 1H), 8.02 (s, 1H), 7.88 (m, 2H), 7.46–7.21 (m, 1H), 7.13 (m, 1H), 7.08–6.67 (m, 3H), 3.99 (m, 3H). ^13^C NMR (176 MHz, DMSO) δ 163.74 (C), 155.93 (C), 153.44 (C), 151.58 (C), 151.02 (C), 145.48 (CH), 136.42 (C), 128.83 (C), 128.23 (2xCH), 124.78 (CH), 123.33 (CH), 121.16 (C), 119.70 (CH), 119.34 (C), 118.41 (CH), 112.55 (2xCH), 106.12 (C), 104.70 (C), 99.20 (CH), 54.86 (CH3). LC-MS *m*/*z* 425.9 [M−H]^−^, 427.9 [M+H]^+^. HPLC (reversed-phase) 0.1% formic acid/Acetonitrile 90:10 to 10:90 in 30 min, λ = 5,282,570 mAU, tR = 20.290 min (98%). Anal. Calcd. for C_22_H_17_N_7_O_3_ (427.14): C, 61.82; H, 4.01; N, 22.94; found C, 61.70; H, 3.96; N, 23.02%.

#### 3.1.2. Preparation of Ethyl 4-((7H-Pyrrolo[2,3-d]pyrimidin-4-yl)amino)benzoate (**7**)

The ethyl 4-aminobenzoate (4.3 gm, 26 mmol) was added to a solution of 4-chloro-7H-pyrrolo[2,3-d]pyrimidine (2 gm, 13 mmol) in absolute ethanol (25 mL). The reaction mixture was stirred at reflux for 7 h. The solid obtained was filtered and washed with cold water to give intermediate **7**. 

White solid, Yield 91.13%; m.p. 243–246 °C; ^1^H NMR (700 MHz, DMSO) δ 11.91 (s, 1H), 9.71 (s, 1H), 8.40 (s, 1H), 8.13 (d, J = 8.4 Hz, 2H), 7.96 (d, J = 8.4 Hz, 2H), 7.32 (s, 1H), 6.89 (s, 1H), 4.68–3.98 (m, 2H), 1.63–0.84 (m, 3H). ^13^C NMR (176 MHz, DMSO) δ 166.18 (C), 153.63 (C), 151.57 (C), 150.93 (CH), 145.59 (C), 130.53 (2xCH), 123.40 (CH), 122.99 (C), 119.19 (2xCH), 104.79 (C), 99.26 (CH), 60.70 (CH2), 14.78 (CH3). LC-MS *m*/*z* 283.1 [M+H]^+^.

#### 3.1.3. Preparation of 4-((7H-Pyrrolo[2,3-d]pyrimidin-4-yl)amino)benzohydrazide (**8**)

Ethyl 4-((7H-pyrrolo[2,3-d]pyrimidin-4-yl)amino)benzoate (1.5 gm, 5 mmol) was reflexed in excess of hydrazine hydrate for 5 h. The mixture was cooled and poured into ice-cold water. The resultant precipitate was filtered, washed thoroughly with water, and dried to obtain intermediate **8**. 

White solid, Yield 76.92%; m.p. > 300 °C; ^1^H NMR (700 MHz, DMSO) δ 11.84 (s, 1H), 9.61 (m, 2H), 8.37 (m, 1H), 8.13–7.92 (m, 2H), 7.85 (m, 2H), 7.29 (s, 1H), 6.85 (s, 1H), 4.46 (s, 2H). ^13^C NMR (176 MHz, DMSO) δ 166.28 (C), 153.59 (C), 151.50 (C), 151.09 (CH), 143.62 (C), 128.05 (2xCH), 126.67 (C), 123.10 (CH), 119.34 (2xCH), 104.53 (C), 99.20 (CH). LC-MS *m*/*z* 268.0 [M−H]^−^, 269.1 [M+H]^+^.

### 3.2. HPLC Analysis

HPLC analysis was carried out using Shimadzu LC-20AD equipped with LC solution software (ver. 5.10.153) and PDA detector (Shimadzu cooperation, Columbia, MD, USA). Analytical Hypersil Nucleosil 250 × 4.6 mm/5 μm C18 column was used as a stationary phase. The mobile phase was 0.1% formic acid and acetonitrile with flow rate of 1 mL/min in a gradient system that began with 10% acetonitrile and changed over 30 min to 90%.

### 3.3. Biological Evaluation

#### 3.3.1. In Vitro Cytotoxic Assay against HepG2, MCF-7, MDA-MB-231, and HeLa Cell Lines

In vitro cytotoxicity assay was assessed against HepG2, MCF-7, MDA-MB-231, and HeLa cell lines, as described in [17,25,26]. Briefly, the cytotoxicity of the synthesized derivatives and the reference compounds was assessed via the MTT assay on four cancer cell lines. The cells were cultured in an RPMI11640 medium with 10% fetal bovine serum (FBS) and an antibiotic cocktail of 100 µL/mL streptomycin and 100 units/mL penicillin. Cancer cell lines were seeded independently in 96-well plates at a concentration of 1.0 × 10^4^ cells/well at 37 °C, 5% CO_2_, and 100% relative humidity for 48 h. Afterwards, the incubated cells were treated with five concentrations of the synthesized derivatives for 24 h. Then, 20 µL MTT (5 mg/mL) was added and left for 4 h before adding 100 µL dimethyl sulfoxide (DMSO) to each well to solubilize the formed formazan. Finally, the absorbance intensity was read at 570 nm using a BioTek EXL 800 plate reader (Agilent Technologies, Inc., Santa Clara, CA, USA). 

#### 3.3.2. Kinase Inhibition Assay over CDK2, EGFR, HER2, and VEGFR2

In vitro kinase inhibition assays were assessed against CDK2, EGFR, HER2, and VEGFR2, as described in [17,27,28]. Briefly, a specific human ELISA kit (Enzyme-Linked Immunosorbent Assay) was used for each kinase enzyme to determine the kinase inhibition activity of compound **5** against EGFR, VEGFR-2, HER2, and CDK2. First, enzymes and specific antibody were placed independently in 96-well plates, and 100 µL of the standard solution or compound **5** was added and left at room temperature for 2.5 h. Afterwards, the wells were washed. Then, 100 µL of the prepared biotin antibody was placed into each well and left for 1 h at room temperature. The wells were washed again before adding 100 µL of streptavidin solution and left for 45 min at room temperature. Then, 100 µL of TMB substrate reagent was added after a third washing step, and the plates were left for 30 min at room temperature. Finally, 50 µL of the stop solution was added to each well and the color intensity was measured at 450 nm.

#### 3.3.3. Cell Cycle Analysis

Effect of compound **5** on cell cycle distribution was analyzed using following the method described in [17,29]. Briefly, to determine the influence of compound **5** on the cell cycle progression of HepG2 cells, flow cytometry analysis was performed using Propidium Iodide flow cytometry kit/BD (ab139418). First, the cells were cultured at a density of 2 × 10^5^/well and incubated for 24 h. Afterwards, the cells were treated with compound 5 for 24 h. Then, the cells were fixed using 70% ethanol for 12 h at 4 °C. The cells were then washed with cold PBS, incubated with 100 µL RNase A for 30 min at 37 °C, and stained with Propidium Iodide (400 µL) in the dark at room temperature for 30 min. The stained cells were identified by running Epics XLMCL™ flow cytometer equipment (Beckman Coulter, Apeldoorn, The Netherlands), and the results were interpreted using Flowing software (version 2.5.1, Turku Centre for Biotechnology, Turku, Finland).

#### 3.3.4. Apoptosis Analysis

Effect of compound **5** on cell programmed cell death was analyzed using Annexin V-FITC cell apoptosis detection kit (K101-100) (BioVision Techology, Inc., Exon, PA, USA) by following the method described in [17,30]. Briefly, in 6-well plates, HepG2 cells at a density of 2 × 10^5^ were cultured and incubated for 24 h. The cultured cells were then treated with compound **5** for 24 h. Afterwards, the cells were trypsinized and gathered via centrifugation (5 min, 300× *g*), washed twice with PBS, and suspended in 0.1 mL of a 1X binding buffer. Then, the cells were double-stained with 5 µL Annexin V-FITC and 5 µL PI in the dark at room temperature for 15 min before they were examined using an Epics XL-MCL™ Flow Cytometer (Beckman Coulter, Apeldoorn, The Netherlands). The excitation wavelength was 488 nm while the emission wavelength was 530 nm. Finally, the data were analyzed using Flowing software (version 2.5.1, Turku Centre for Biotechnology, Turku, Finland).

#### 3.3.5. Caspase 3, Caspase 9, BAX, and Bcl-2 Level Protein Assays

Compound 5 effect on Caspase 3, caspase 9, BAX, and Bcl-2 levels was analyzed by following the method described in [17]. In 96-well plates, HepG2 cells were cultured and incubated for 24 h. The cells were then treated with compound **5** or 0.1% DMSO (*v*/*v*) for 24 h. Afterwards, caspase 3 and 9, BAX, and Bcl-2 levels were determined using ELISA assay kits KHO1091 (InvitrogenTM, Grand Island, NY, USA), EIA-4860 (DRU International Inc., Mountainside, NJ, USA), EIA-4487 (DRU International Inc., Mountainside, NJ, USA), and 99-0042 (InvitrogenTM, Grand Island, NY, USA), respectively, according to the manufacturers’ procedures.

### 3.4. Molecular Docking

Molecular docking studies of synthesized compounds docked into the active sites of selected protein kinases were performed using AutoDock Vina and PyRx (The Scripps Research Institute, La Jolla, CA, USA) software (0.8) and visualized by Discovery studio visualizer, as described in [17,19,31].

### 3.5. ADMET Profile Study

ADMET profiles of the new synthesized compound **1**–**5** and sunitinib were in silico predicted by following pkCSM algorithm protocol [24].

## 4. Conclusions

Kinase enzymes have been a target for anticancer agents due to their role in the regulation of cancer. Successfully, five new derivatives of 7-deazapurine incorporating compounds were designed, synthesized, and biologically evaluated. All compounds were investigated for cytotoxicity effects on four cancer cell lines. The most active candidate, compound **5**, showed promising results in further biological investigation, such as EGFR, Her2, VEGFR2, and CDK2 protein kinases inhibition assays, cell cycle analysis, apoptosis, and caspase 3 and caspase 9, Bax, and Bcl-2. Molecular docking studies demonstrated that compound **5** superimposed the crystallized ligands of the active sites of the selected kinase enzymes and had almost similar binding interactions. Overall, compound **5** was reported in this study as a promising multikinase inhibitor, which needs more mechanistic investigation to determine the actual mechanism of the anticancer effect. 

## Figures and Tables

**Table 1:** Physicochemical properties and ADMET profiles of isatin–deazapurine hybrid compounds **1**–**5**, calculated by pkCSM.

Parameters		1	2	3	4	5	Sunitinib
	Compound						
Molecular properties							
Molecular weight		397.398	431.843	415.388	411.425	427.424	398.482
LogP		2.7877	3.4411	2.9268	3.0961	2.7963	3.33494
H-acceptor		6	6	6	6	7	3
H-donors		4	4	4	4	4	3
Surface area		169.803	180.106	173.968	176.168	181.281	169.730
Absorption							
Water solubility		−3.118	−3.122	−3.117	−3.127	−3.116	−4.338
Intestinal absorption (human)		79.999%	81.175%	78.38%	80.467%	76.475%	94.534%
Skin permeability		−2.735	−2.735	−2.735	−2.735	−2.735	−2.79
Distribution							
BBB permeability		−1.325	−1.507	−1.486	−1.354	−1.476	−1.122
CNS permeability		−2.474	−2.367	−2.541	−2.407	−2.667	−2.601
Metabolism							
CYP1A2 inhibitor		YES	**YES**	**YES**	YES	**YES**	NO
CYP2C19 inhibitor		YES	YES	YES	YES	YES	NO
CYP2C9 inhibitor		YES	YES	YES	YES	YES	NO
CYP2D6 inhibitor		NO	NO	NO	NO	NO	YES
CYP3A4 inhibitor		YES	YES	YES	YES	YES	YES
Excretion							
Total clearance		−0.022	−0.281	0.08	−0.018	0.186	1.055
Renal OCT2 substrate		NO	NO	NO	NO	NO	NO
Toxicity							
Max. tolerated dose (human)		0.587	0.626	0.621	0.616	0.635	0.223
Oral rate acute toxicity (LD50)		2.837	2.528	2.773	2.854	2.776	2.387
Oral rate chronic toxicity (LOAEL)		2.651	2.528	2.363	2.481	2.314	1.662
Hepatotoxicity		YES	YES	YES	YES	YES	YES
Skin sensitization		NO	NO	NO	NO	NO	NO

**Table 2:** Cytotoxicity assay results of isatin–deazapurine hybrid compounds **1**–**5**.

Compound	R	In Vitro Cytotoxicity IC_50_ (µM)			
		HepG2	MCF-7	MDA-MB-231	HeLa
**1**	H	25.78 ± 1.9	18.49 ± 1.5	16.03 ± 1.3	12.39 ± 0.9
**2**	Cl	67.42 ± 3.6	72.51 ± 3.7	51.29 ± 2.9	45.40 ± 2.6
**3**	F	59.31 ± 3.3	63.68 ± 3.4	42.85 ± 2.6	39.15 ± 2.4
**4**	CH_3_	92.67 ± 4.5	80.23 ± 4.2	73.22 ± 3.3	75.36 ± 3.5
**5**	**OCH_3_**	**6.11 ± 0.4**	**5.93 ± 0.3**	**2.48 ± 0.1**	**1.98 ± 0.1**
**DOXORUBICIN**	-	4.50 ± 0.2	4.17 ± 0.2	3.18 ± 0.1	5.57 ± 0.4
**Sunitinib**	-	6.82 ± 0.5	5.19 ± 0.4	8.41 ± 0.7	7.48 ± 0.6

All IC_50_ values are presented as the mean ± SD of three different experiments.

**Table 3:** Results of inhibitory effects against EGFR, HER2, VEGFR2, and CDK2 induced by compound **5**.

IC_50_ (μM)	CDK2	EGFR	HER2	VEGFR2
**Compound 5**	0.131 ± 0.007	0.103 ± 0.006	0.081 ± 0.002	0.178 ± 0.009
**Reference**	* 0.063 ± 0.003	** 0.041 ± 0.003	*** 0.06 ± 0.006	**** 0.045 ± 0.002

All IC_50_ values are presented as the mean ± SD of three different experiments. References: * Ribocicli, ** Erlotinib, *** Lapatinib, **** Sorafenib.

**Table 4:** Compound **5** effect on the different phases of cell cycle of HepG2 cells.

Cell Line	Cell Cycle Distribution (%)			
	%G0-G1	%S	%G2/M	%Pre-G1
Compound **5**	49.02	44.93	6.05	39.72
Cont. HepG2	43.97	41.12	14.91	1.86

All percentages are presented as mean of three different experiments.

**Table 5:** Compound **5** apoptosis effect in HepG2 cells.

Sample	Apoptosis			Necrosis
	Viable	Early	Late	
Compound **5**/HepG2	39.72	13.28	21.16	5.28
Cont. HepG2	1.86	0.62	0.13	1.11

All percentages are presented as mean of three different experiments.

**Table 6:** Compound **5** effects on levels of caspase 3, caspase 9, Bax, and Bcl-2.

Sample	Gene Expression Fold Change			
	Caspase 3	Caspase 9	Bax	Bcl-2
Compound **5**/HepG2	7.984	4.853	6.13	0.184
Cont. HepG2	1	1	1	1

## Data Availability

Not applicable.

## Supplementary Materials

The following supporting information can be downloaded at: https://www.mdpi.com/article/10.3390/molecules28155869/s1. Table S1: Physicochemical properties and ADMET profiles of isatin-deazapurine hybrid compounds **1**–**5** and **9**–**12**, calculated by pkCSM; Figure S1: 3D and 2D interactions of compound **1** in the active site of CDK2; Figure S2: 3D and 2D interactions of compound **2** in the active site of CDK2; Figure S3: 3D and 2D interactions of compound **3** in the active site of CDK2; Figure S4: 3D and 2D interactions of compound **4** in the active site of CDK2.

## Sample Availability

Samples of the compounds are not available from the authors.
