# Supplementary material for: Novel 7-Deazapurine Incorporating Isatin Hybrid Compounds as Protein Kinase Inhibitors: Design, Synthesis, In Silico Studies, and Antiproliferative Evaluation
Source: Molecules. 2023 Aug 4;28(15):5869. doi: 10.3390/molecules28155869 (PMC10420662; doi:10.3390/molecules28155869)
Supplement: Supplementary file 1 [file molecules-28-05869-s001.zip › molecules-2476875-supplementary.pdf]

Novel 7-Deazapurine Incorporating Isatin Hybrid Compounds as Protein Kinase Inhibitors: Design, Synthesis, In Silico Studies, and Antiproliferative Evaluation

Mohammed M. Alanazi [1,*,†] and Ashwag S. Alanazi [2,*,†]

[1] Department of Pharmaceutical Chemistry, College of Pharmacy, King Saud University, P.O. Box 2457, Riyadh 11451, Saudi Arabia
[2] Department of Pharmaceutical Sciences, College of Pharmacy, Princess Nourah Bint Abdulrahman University, Riyadh 11671, Saudi Arabia
* Correspondence: mmalanazi@ksu.edu.sa (M.M.A.); asalanzi@pnu.edu.sa (A.S.A.)
† These authors contributed equally to this work.

1. Chemistry:

1.1. Compound 1

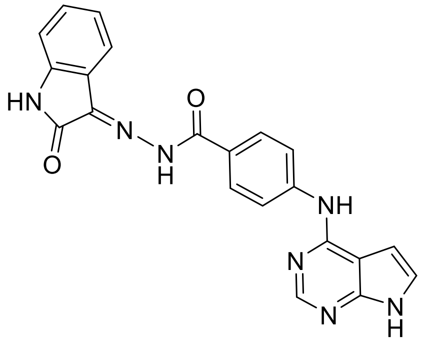

4-((7*H*-pyrrolo[2,3-*d*]pyrimidin-4-yl)amino)-*N*'-(2-oxoindolin-3-ylidene)benzohydrazide

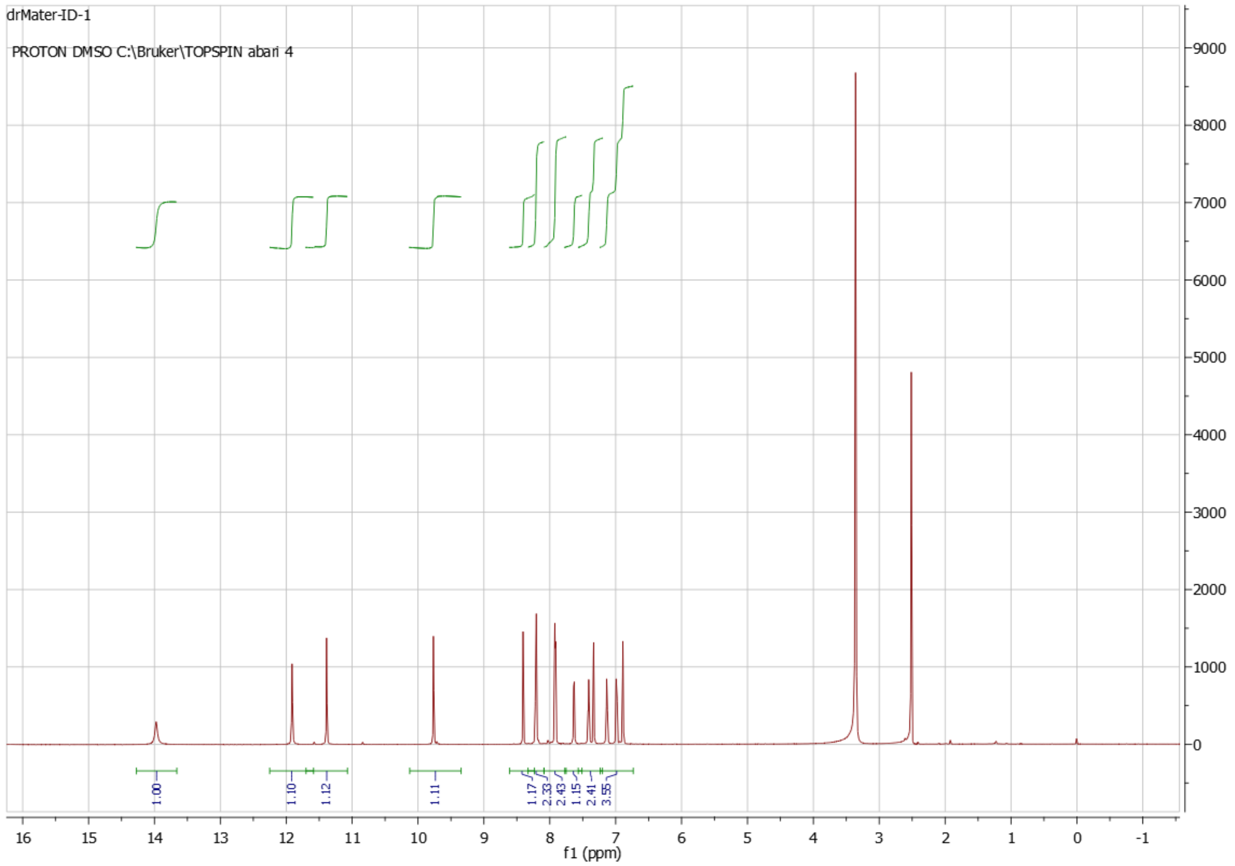
drMater-ID-1
PROTON DMSO C:\Bruker\TOPSPIN abari 4
1.00
1.10
1.12
1.11
1.17
2.33
2.43
1.15
2.41
3.55
f1 (ppm)

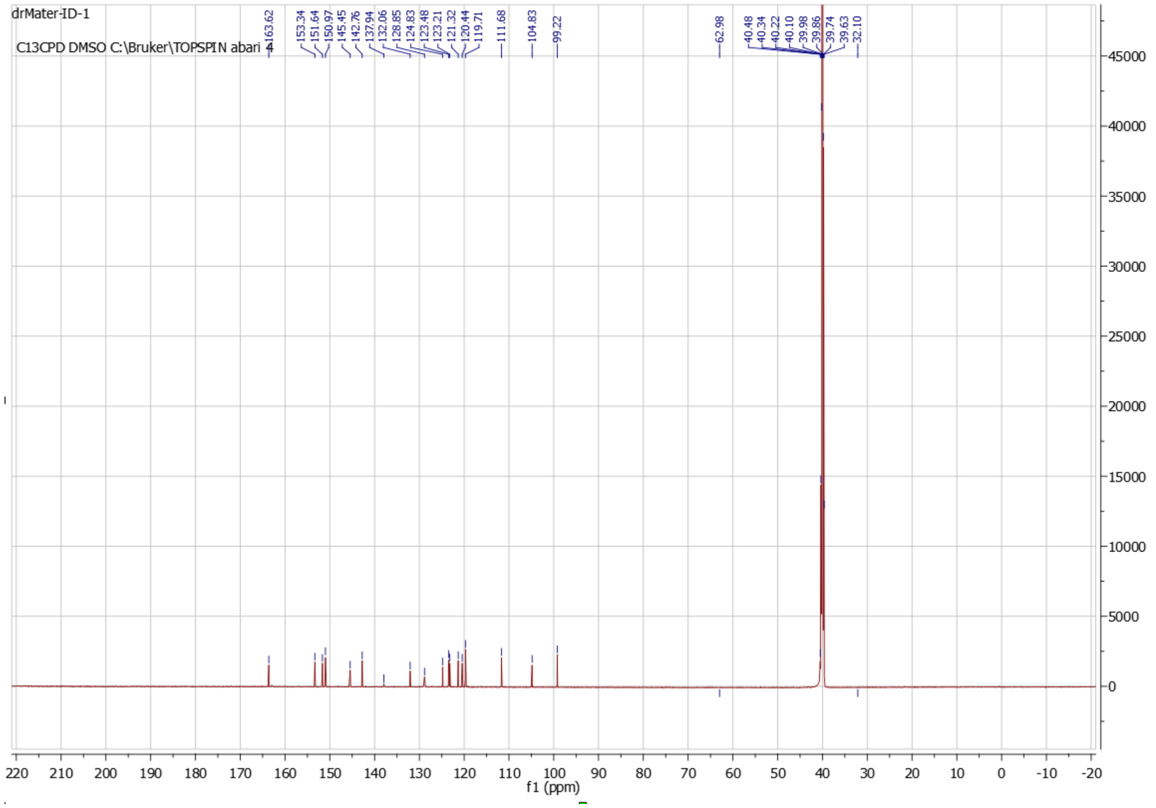
drMater-ID-1
C13CPD DMSO C:\Bruker\TOPSPIN abari 4
163.62
153.34
151.64
150.97
145.45
142.76
137.94
132.06
128.85
124.83
123.48
123.21
121.32
120.44
119.71
111.68
104.83
99.22
62.98
40.48
40.34
40.22
40.10
39.98
39.86
39.74
39.63
32.10
f1 (ppm)

LCMS negative

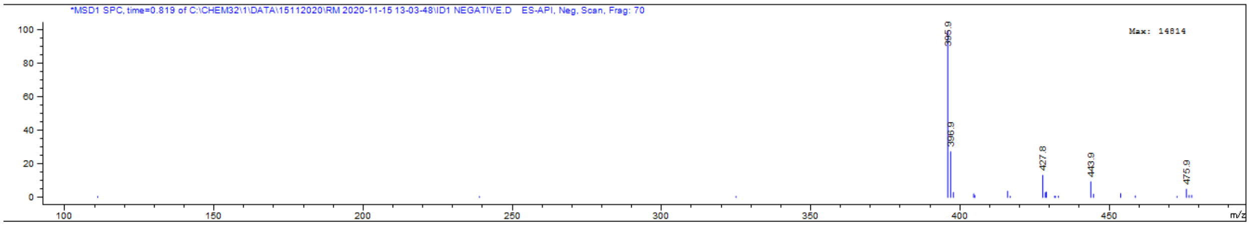

LCMS positive

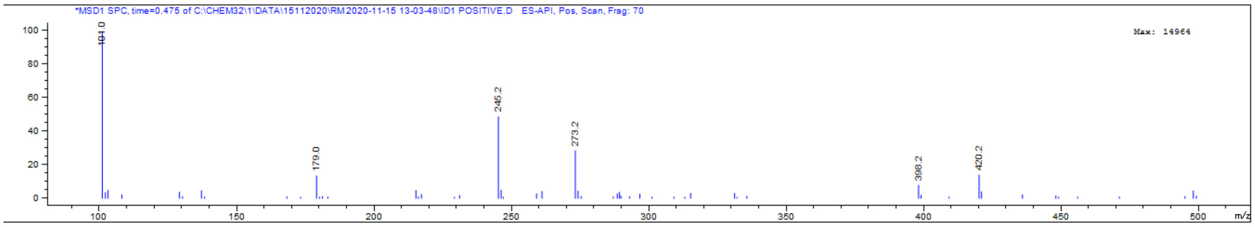

HPLC

==== Shimadzu LCsolution Analysis Report ====

C:\LabSolutions\Data\AWADH\TKI-1-10.lcd

Acquired by	: Admin
Sample Name	: TKI-1
Sample ID	: TKI-1-10
Tray#	: 1
Vail #	: 1
Injection Volume	: 10 uL
Data File Name	: TKI-1-10.lcd
Method File Name	: Awadh-purity.lcm
Batch File Name	: AWADH-WASH.lcb
Report File Name	: Default.lcr
Data Acquired	: 7/17/2023 10:53:37 AM
Data Processed	: 7/18/2023 9:04:12 AM

<Chromatogram>

C:\LabSolutions\Data\AWADH\TKI-1-10.lcd

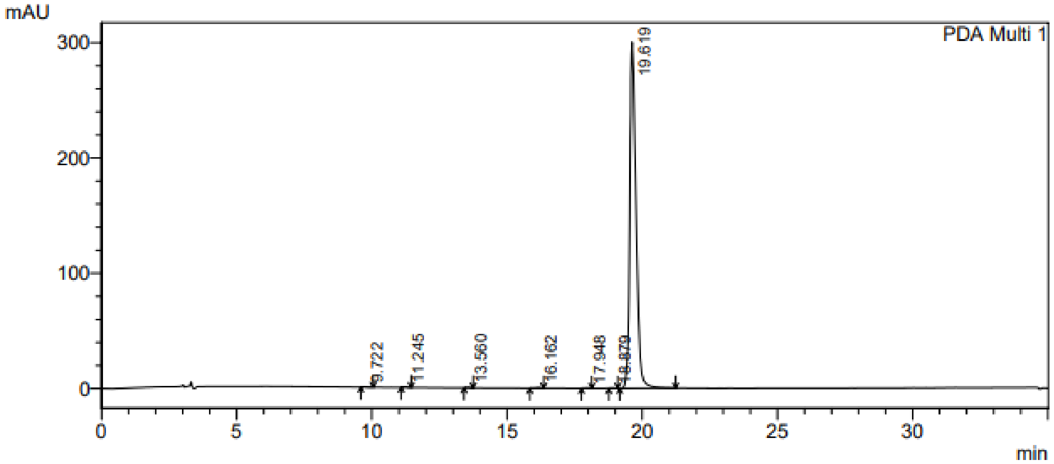

1 PDA Multi 1/318nm 4nm

PeakTable

PDA Ch1 318nm 4nm

Peak#	Ret. Time	USP Width	Area	Height	Area %	Height %
1	9.722	0.207	2899	391	0.061	0.129
2	11.245	0.223	4315	558	0.090	0.184
3	13.560	0.202	3499	511	0.073	0.169
4	16.162	0.264	5500	516	0.115	0.170
5	17.948	0.251	2534	283	0.053	0.094
6	18.879	0.283	3314	348	0.069	0.115
7	19.619	0.424	4767400	300314	99.539	99.139
Total			4789461	302921	100.000	100.000

1.2. Compound 2

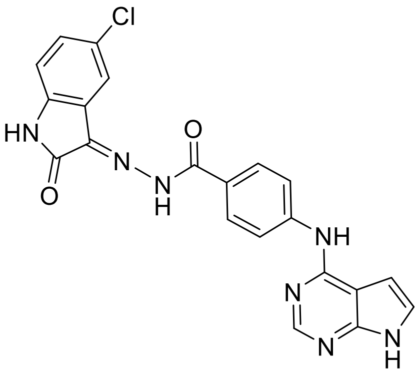

4-((7*H*-pyrrolo[2,3-*d*]pyrimidin-4-yl)amino)-*N*'-(5-chloro-2-oxoindolin-3-ylidene)benzohydrazide

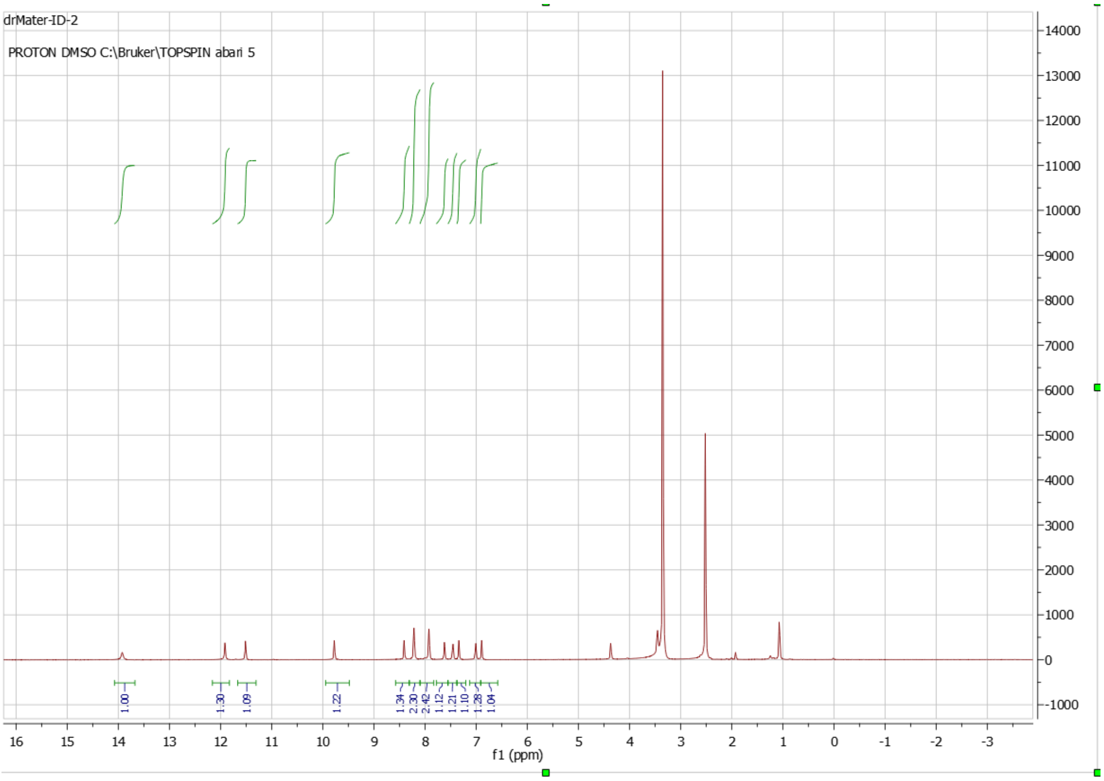

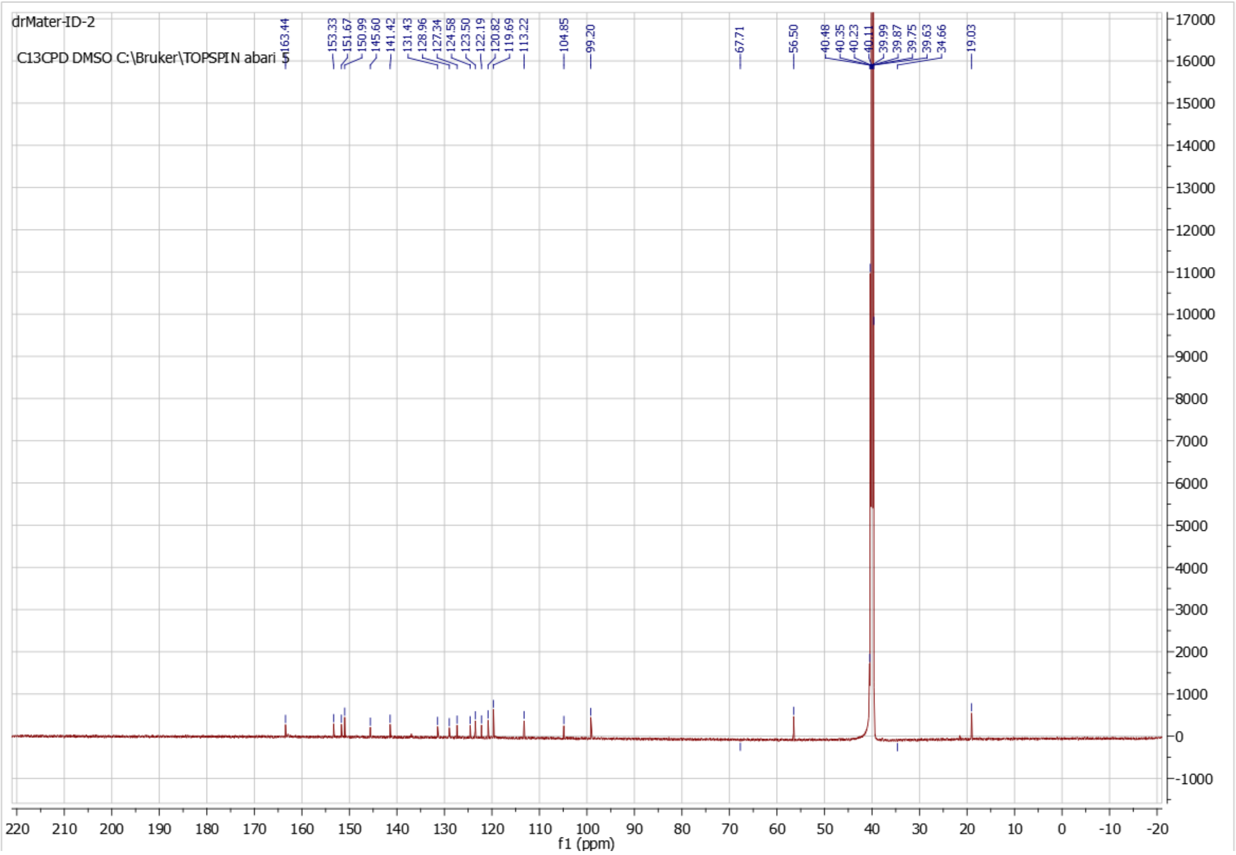

LCMS negative

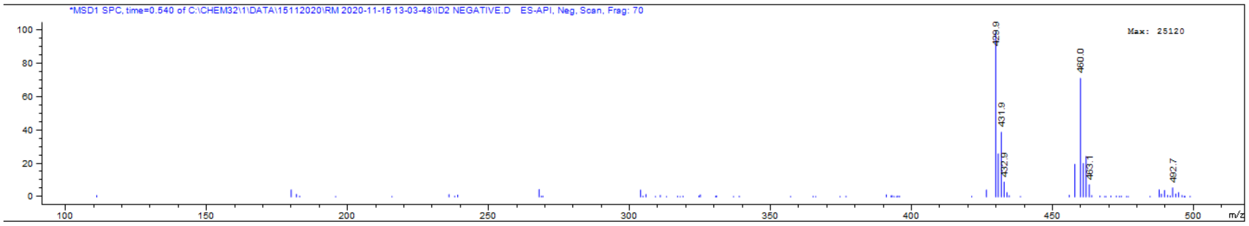

HPLC

==== Shimadzu LCsolution Analysis Report ====

C:\LabSolutions\Data\AWADH\TKI-1-20.lcd

Acquired by	: Admin
Sample Name	: TKI-2
Sample ID	: TKI-1-20
Tray#	: 1
Vail #	: 2
Injection Volume	: 10 uL
Data File Name	: TKI-1-20.lcd
Method File Name	: Awadh-purity.lcm
Batch File Name	: AWADH-WASH.lcb
Report File Name	: Default.lcr
Data Acquired	: 7/17/2023 11:29:10 AM
Data Processed	: 7/18/2023 9:09:35 AM

<Chromatogram>

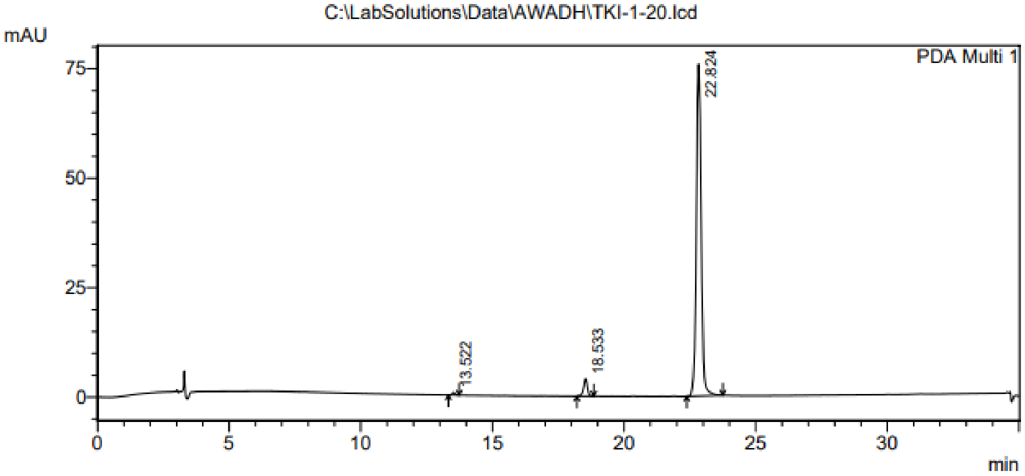

1 PDA Multi 1/360nm 4nm

PeakTable

PDA Ch1 360nm 4nm

Peak#	Ret. Time	USP Width	Area	Height	Area %	Height %
1	13.522	0.213	4493	586	0.447	0.730
2	18.533	0.262	38718	3931	3.854	4.898
3	22.824	0.330	961476	75735	95.699	94.372
Total			1004687	80252	100.000	100.000

1.3. Compound 3

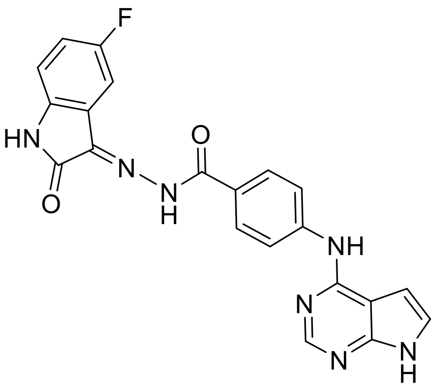

4-((7*H*-pyrrolo[2,3-*d*]pyrimidin-4-yl)amino)-*N*'-(5-fluoro-2-oxoindolin-3-ylidene)benzohydrazide

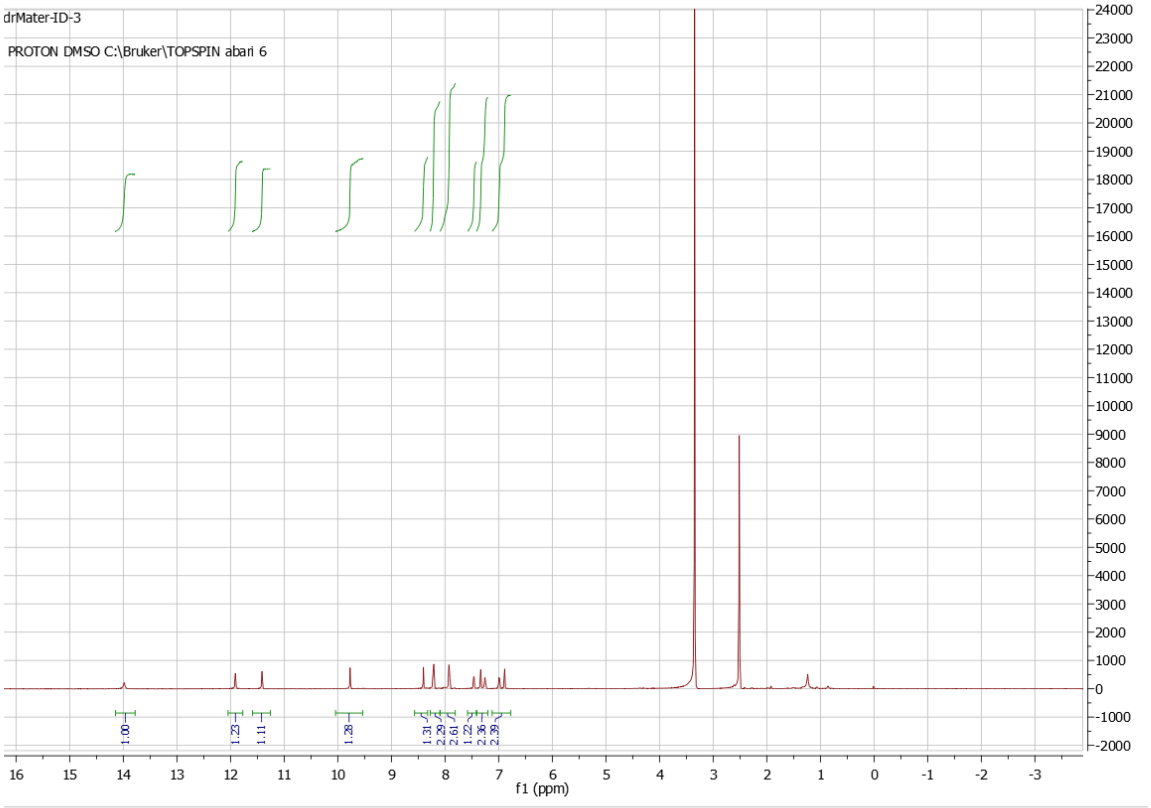

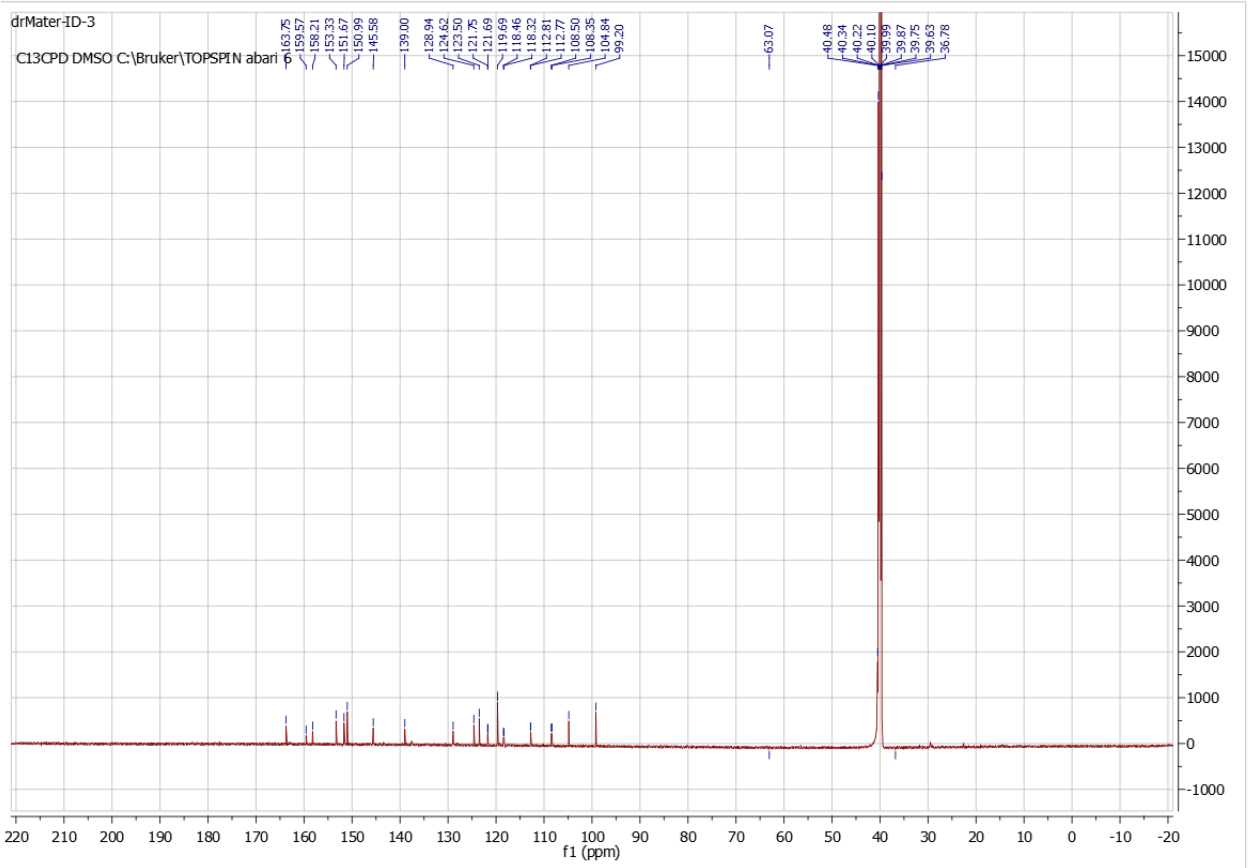

LCMS negative

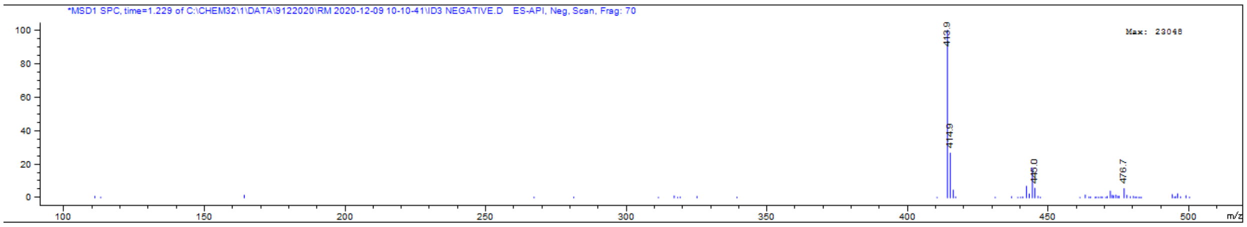

HPLC

==== Shimadzu LCsolution Analysis Report ====

C:\LabSolutions\Data\AWADH\TKI-1-30.lcd

Acquired by	: Admin
Sample Name	: TKI-3
Sample ID	: TKI-1-30
Tray#	: 1
Vail #	: 3
Injection Volume	: 10 uL
Data File Name	: TKI-1-30.lcd
Method File Name	: Awadh-purity.lcm
Batch File Name	: AWADH-WASH.lcb
Report File Name	: Default.lcr
Data Acquired	: 7/17/2023 12:04:44 PM
Data Processed	: 7/18/2023 9:11:41 AM

<Chromatogram>

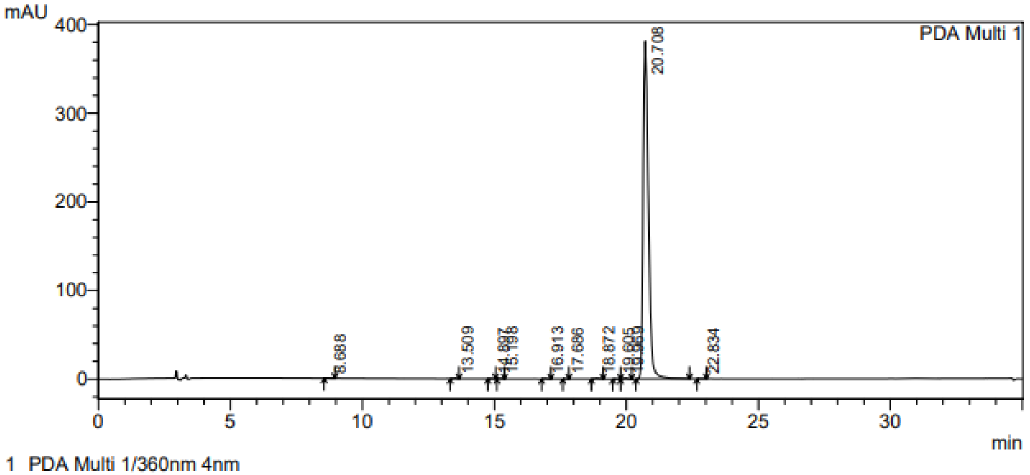

1 PDA Multi 1/360nm 4nm

PeakTable

PDA Ch1 360nm 4nm

Peak#	Ret. Time	USP Width	Area	Height	Area %	Height %
1	8.688	0.263	2487	263	0.047	0.068
2	13.509	0.204	5035	706	0.096	0.183
3	14.897	0.242	1196	152	0.023	0.039
4	15.198	0.191	2551	384	0.049	0.100
5	16.913	0.206	3597	499	0.068	0.130
6	17.686	0.210	1053	143	0.020	0.037
7	18.872	0.265	7849	845	0.149	0.219
8	19.605	0.323	1179	103	0.022	0.027
9	19.969	0.264	8933	927	0.170	0.240
10	20.708	0.368	5218874	381102	99.315	98.897
11	22.834	0.260	2100	227	0.040	0.059
Total			5254856	385351	100.000	100.000

1.4. Compound 4

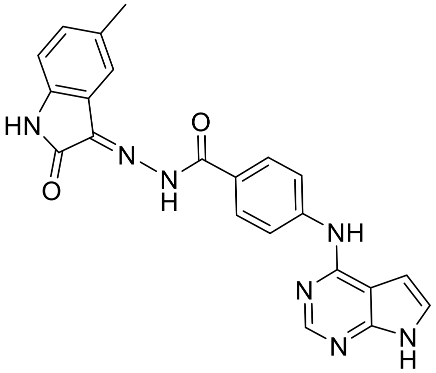

4-((7*H*-pyrrolo[2,3-*d*]pyrimidin-4-yl)amino)-*N*'-(5-methyl-2-oxoindolin-3-ylidene)benzohydrazide

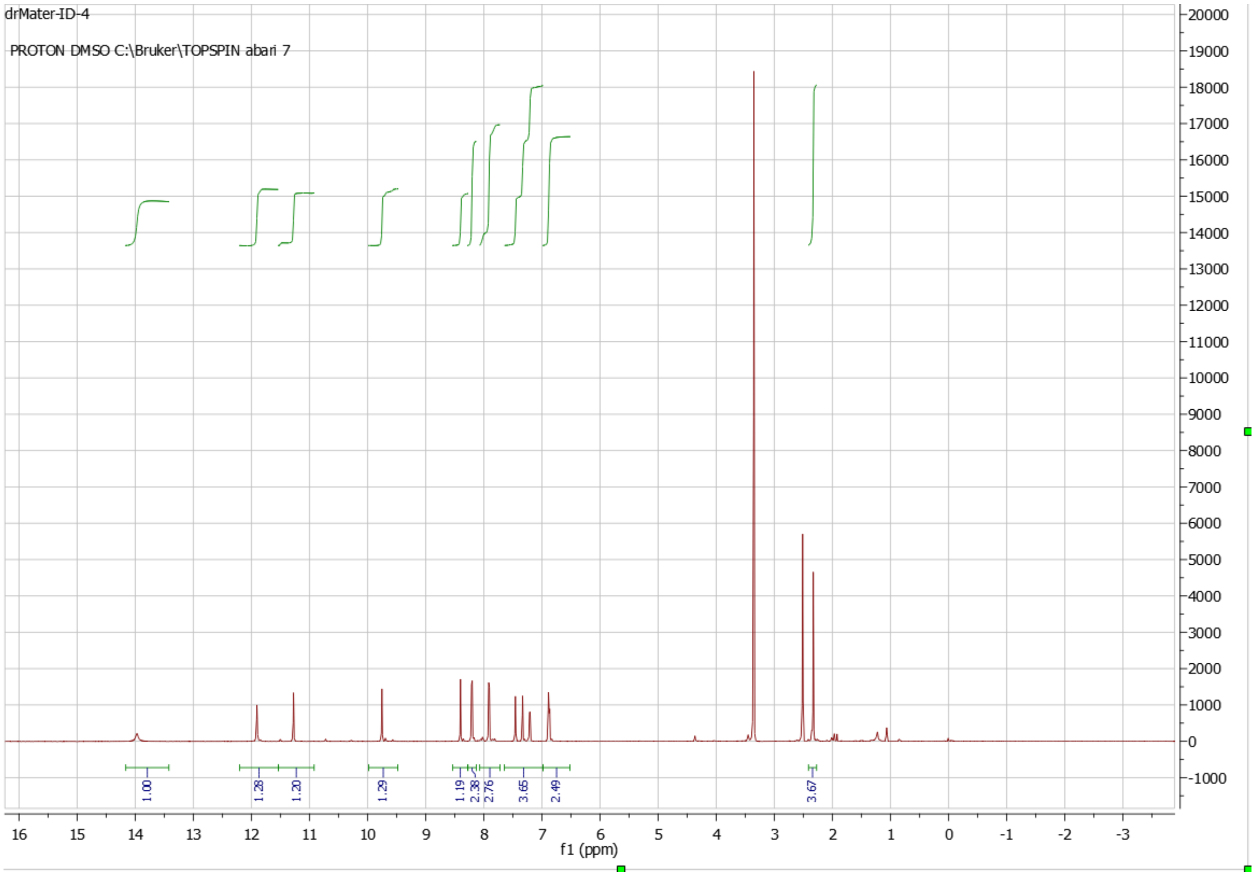

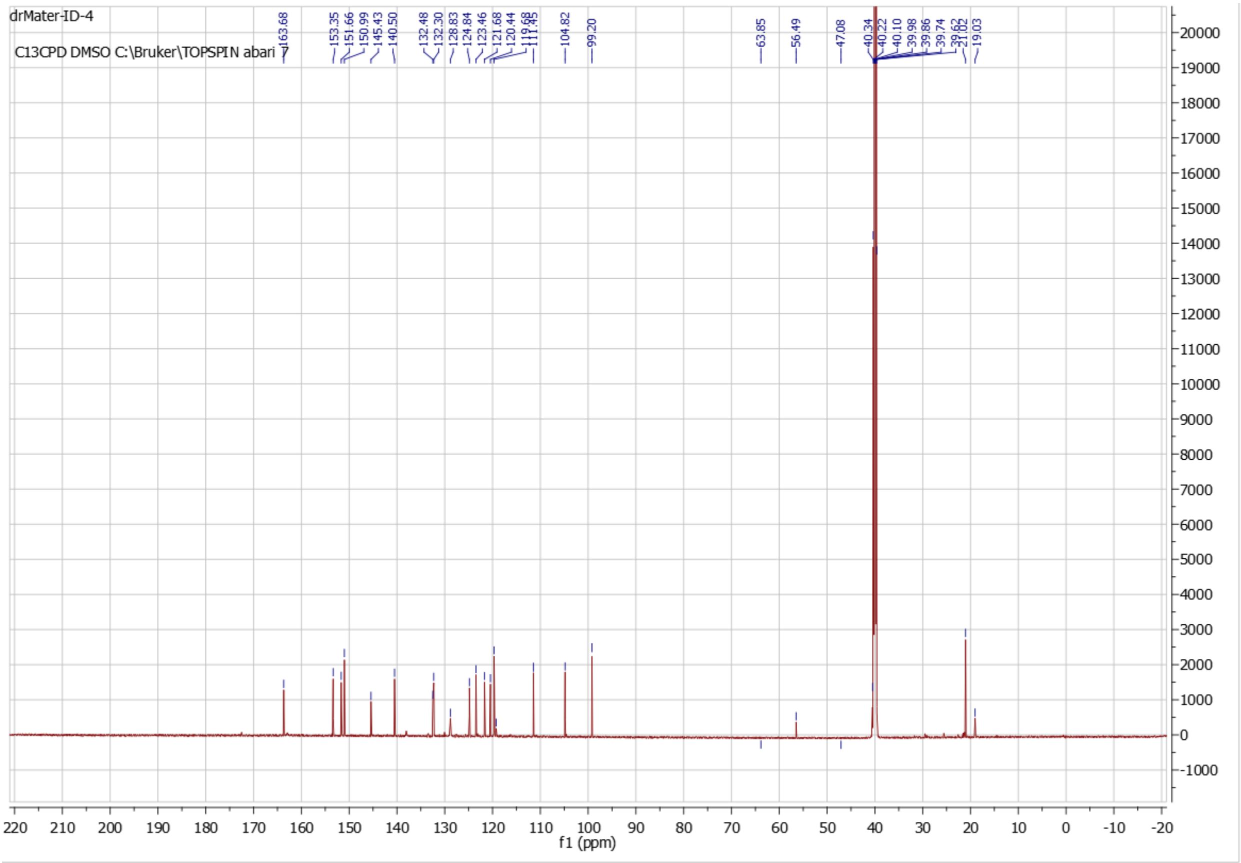

LCMS negative

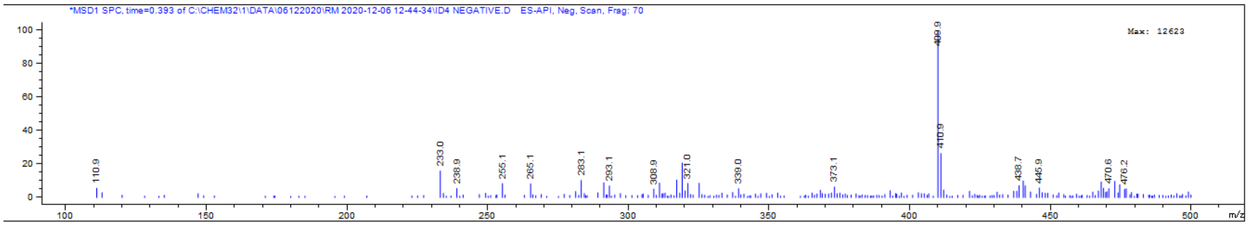

HPLC

==== Shimadzu LCsolution Analysis Report ====

C:\LabSolutions\Data\AWADH\TKI-1-40.lcd

Acquired by	: Admin
Sample Name	: TKI-4
Sample ID	: TKI-1-40
Tray#	: 1
Vail #	: 4
Injection Volume	: 10 uL
Data File Name	: TKI-1-40.lcd
Method File Name	: Awadh-purity.lcm
Batch File Name	: AWADH-WASH.lcb
Report File Name	: Default.lcr
Data Acquired	: 7/17/2023 12:40:13 PM
Data Processed	: 7/18/2023 9:13:27 AM

<Chromatogram>

C:\LabSolutions\Data\AWADH\TKI-1-40.lcd

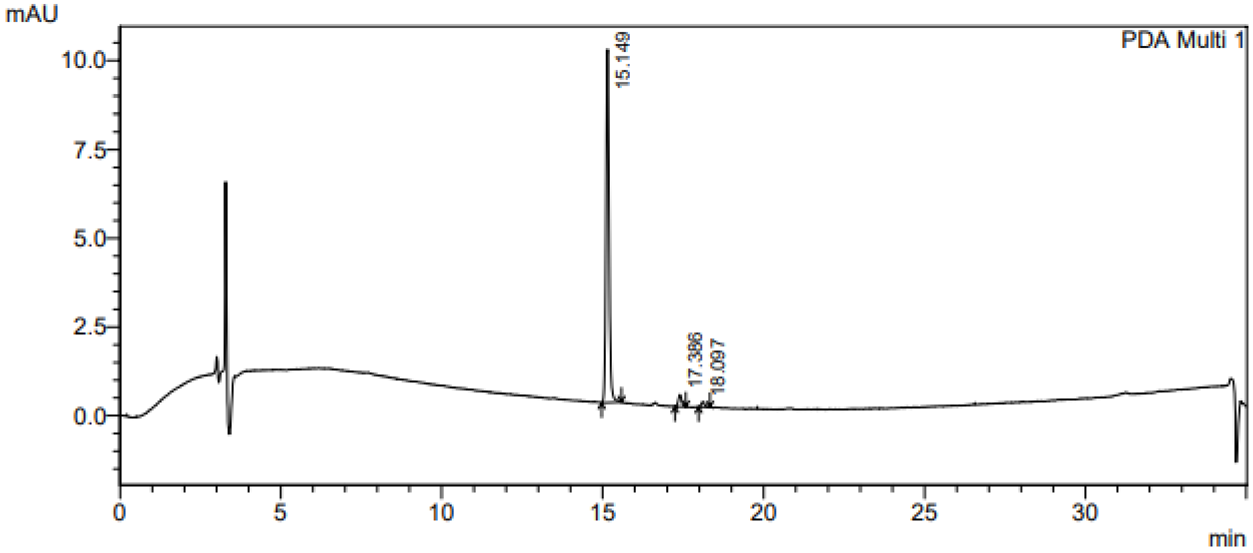

1 PDA Multi 1/360nm 4nm

PeakTable

PDA Ch1 360nm 4nm

Peak#	Ret. Time	USP Width	Area	Height	Area %	Height %
1	15.149	0.191	66504	9945	94.914	95.300
2	17.386	0.201	2467	340	3.521	3.259
3	18.097	0.208	1097	150	1.565	1.442
Total			70068	10436	100.000	100.000

1.5. Compound 5

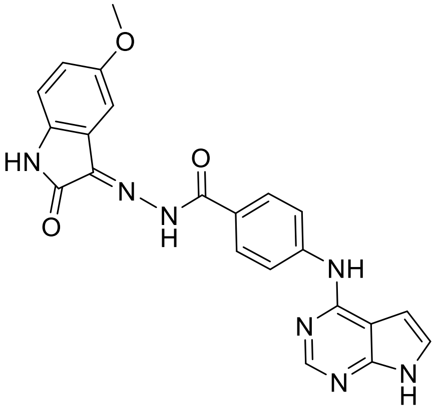

4-((7*H*-pyrrolo[2,3-*d*]pyrimidin-4-yl)amino)-*N*'-(5-methoxy-2-oxoindolin-3-ylidene)benzohydrazide

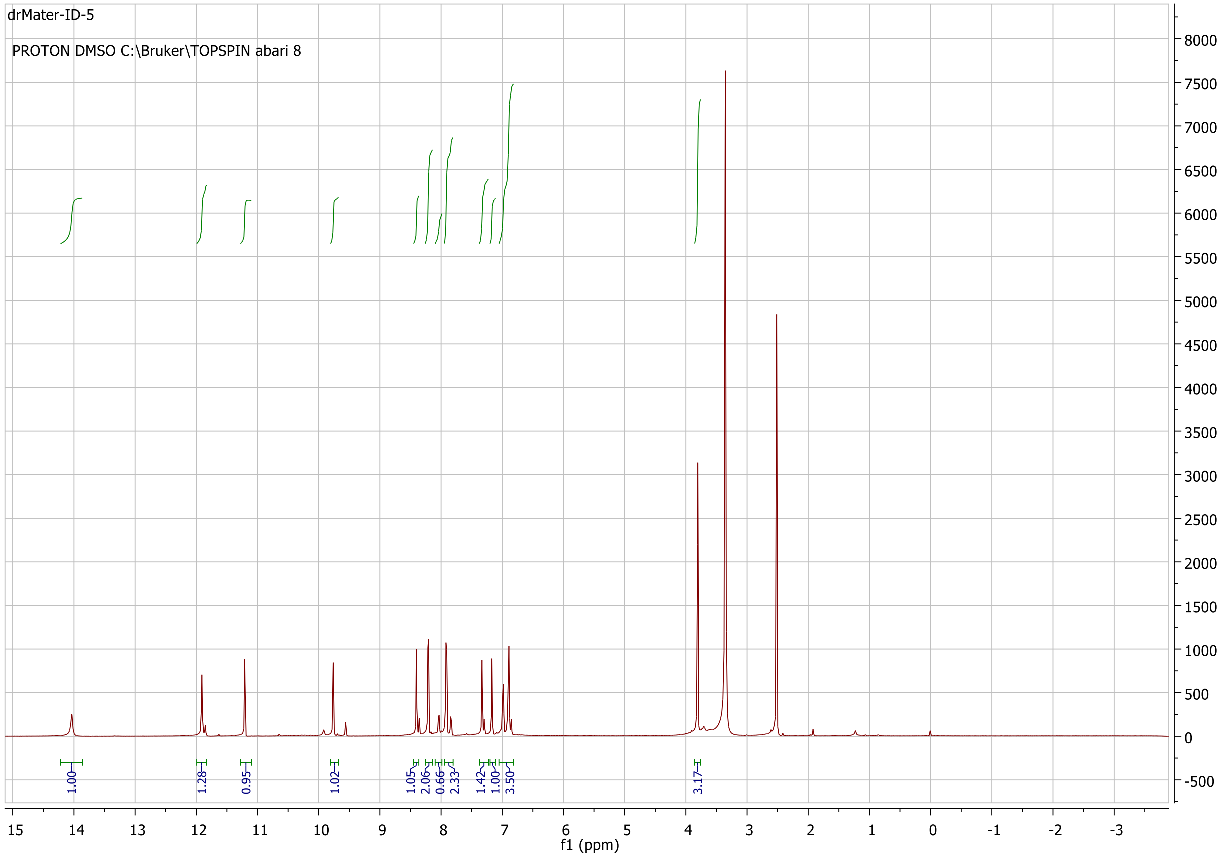

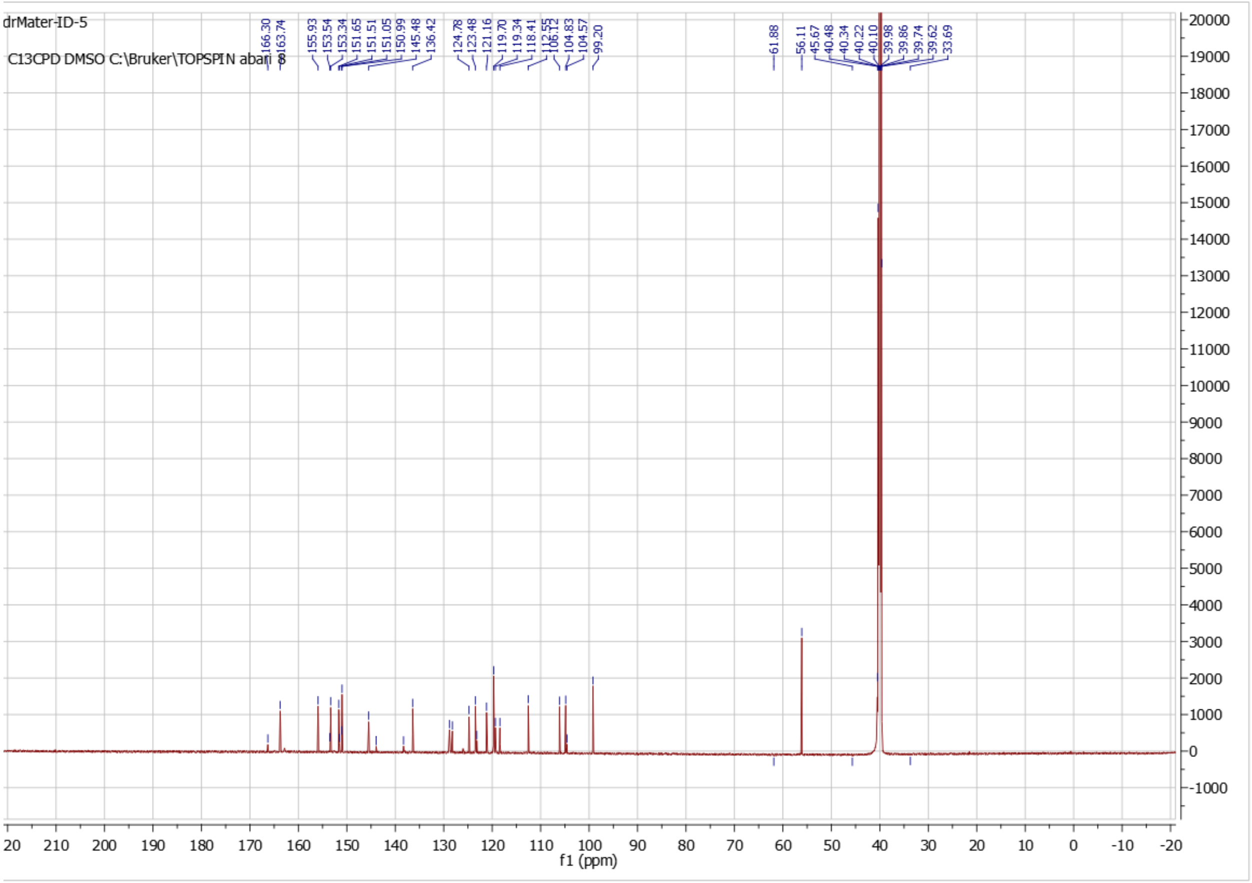

LCMS negative

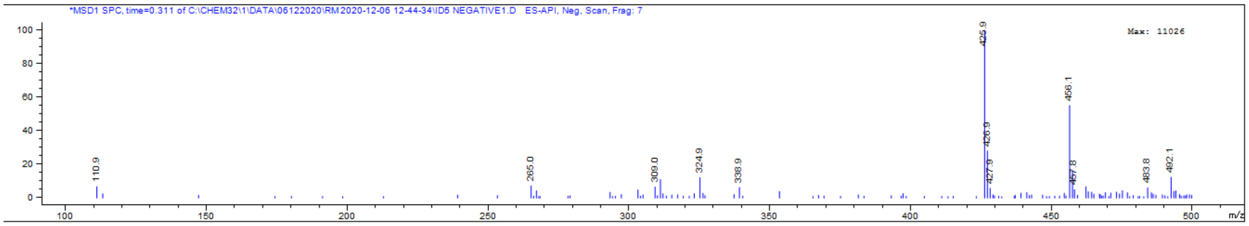

HPLC

==== Shimadzu LCsolution Analysis Report ====

C:\LabSolutions\Data\AWADH\TKI-1-50.lcd

Acquired by	: Admin
Sample Name	: TKI-5
Sample ID	: TKI-1-40
Tray#	: 1
Vail #	: 5
Injection Volume	: 10 uL
Data File Name	: TKI-1-50.lcd
Method File Name	: Awadh-purity.lcm
Batch File Name	: AWADH-WASH.lcb
Report File Name	: Default.lcr
Data Acquired	: 7/17/2023 1:15:44 PM
Data Processed	: 7/18/2023 9:17:19 AM

<Chromatogram>

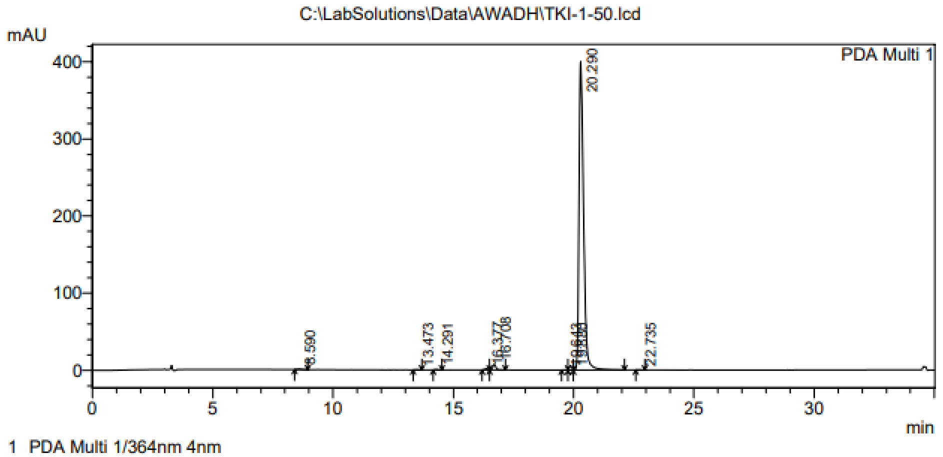

1 PDA Multi 1/364nm 4nm

PeakTable

PDA Ch1 364nm 4nm

Peak#	Ret. Time	USP Width	Area	Height	Area %	Height %
1	8.590	0.277	7321	713	0.136	0.173
2	13.473	0.222	2235	295	0.042	0.072
3	14.291	0.219	5569	713	0.103	0.173
4	16.377	0.237	14183	1705	0.263	0.415
5	16.708	0.242	66873	7393	1.242	1.798
6	19.643	0.237	1160	141	0.022	0.034
7	19.880	0.223	1171	159	0.022	0.039
8	20.290	0.355	5282570	399757	98.130	97.237
9	22.735	0.256	2159	238	0.040	0.058
Total			5383241	411115	100.000	100.000

1.6. Sunitinib

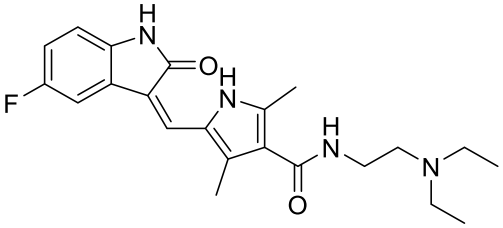

Physicochemical properties and DMET profiles of sunitinib, calculated by pkCSM: https://biosig.lab.uq.edu.au/pkcsm/prediction_single/adme_1683990780.92

1.7. Compound 7

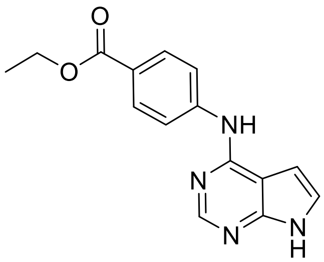

ethyl 4-((7*H*-pyrrolo[2,3-*d*]pyrimidin-4-yl)amino)benzoate

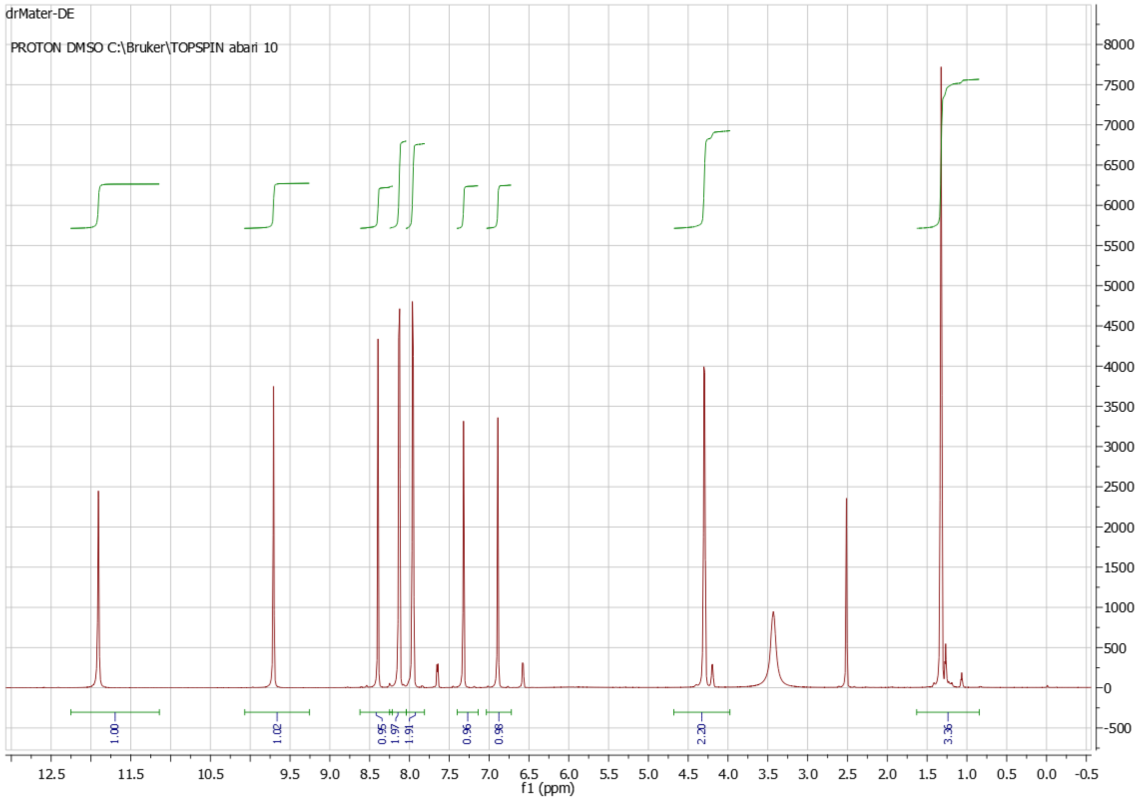

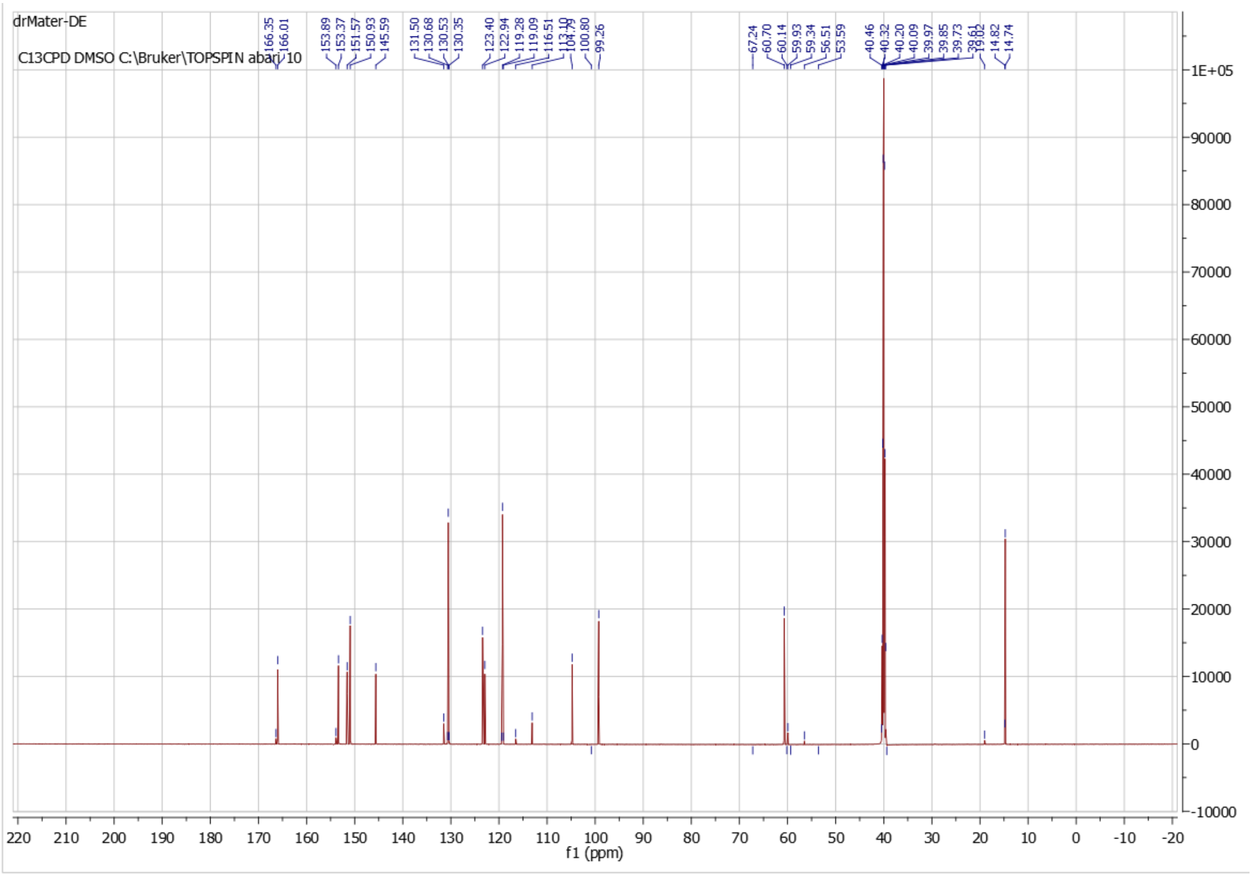

LCMS Positive

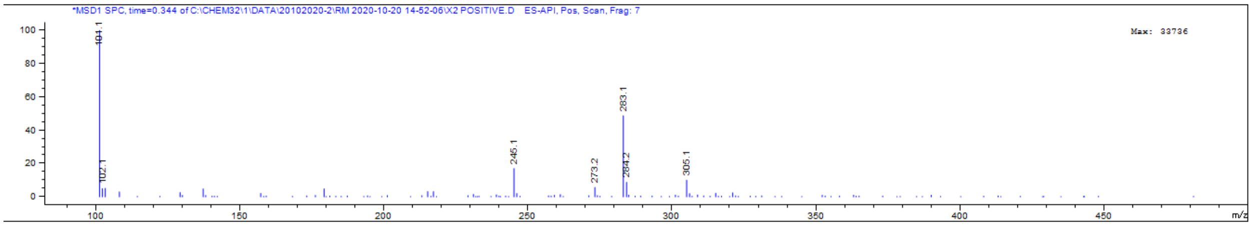

1.8. Compound 8

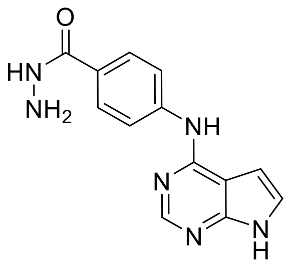

4-((7*H*-pyrrolo[2,3-*d*]pyrimidin-4-yl)amino)benzohydrazide

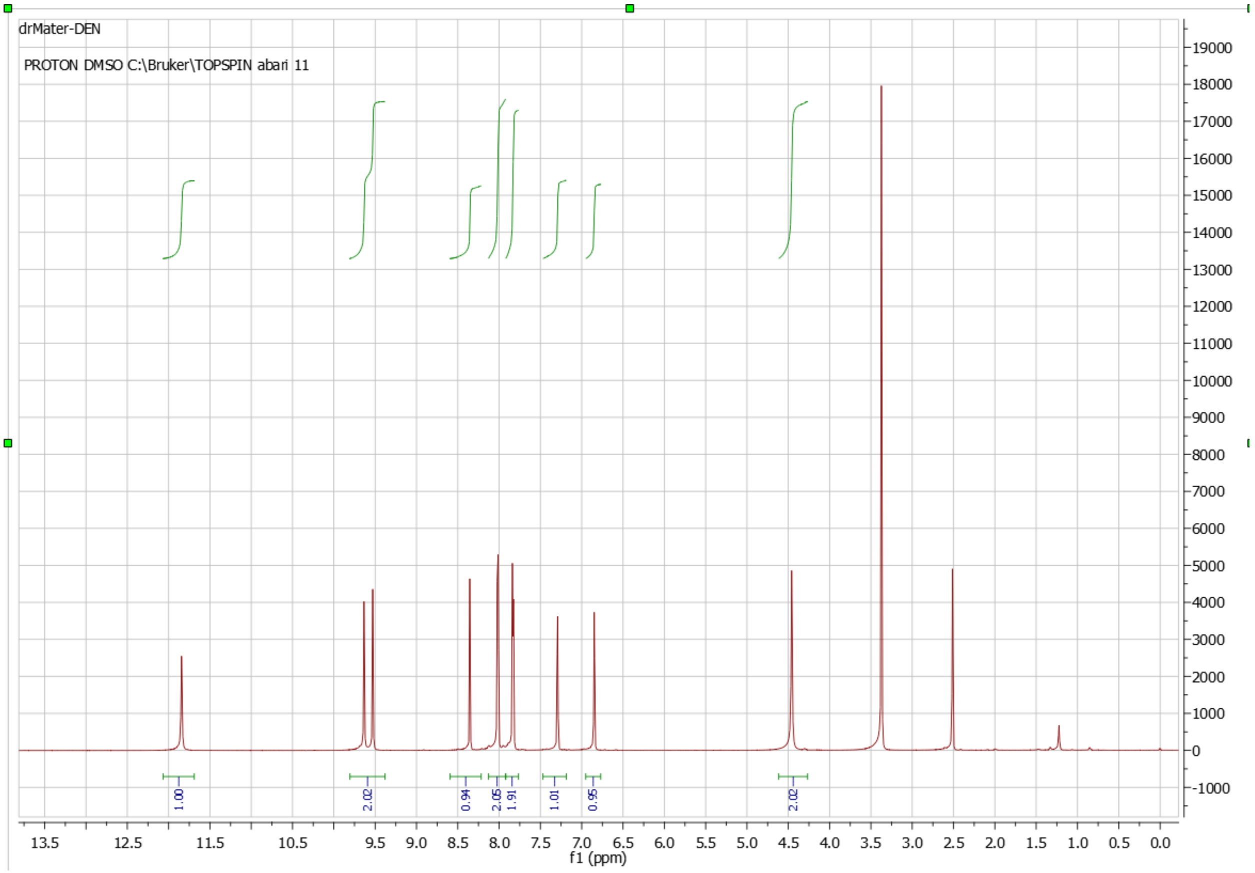

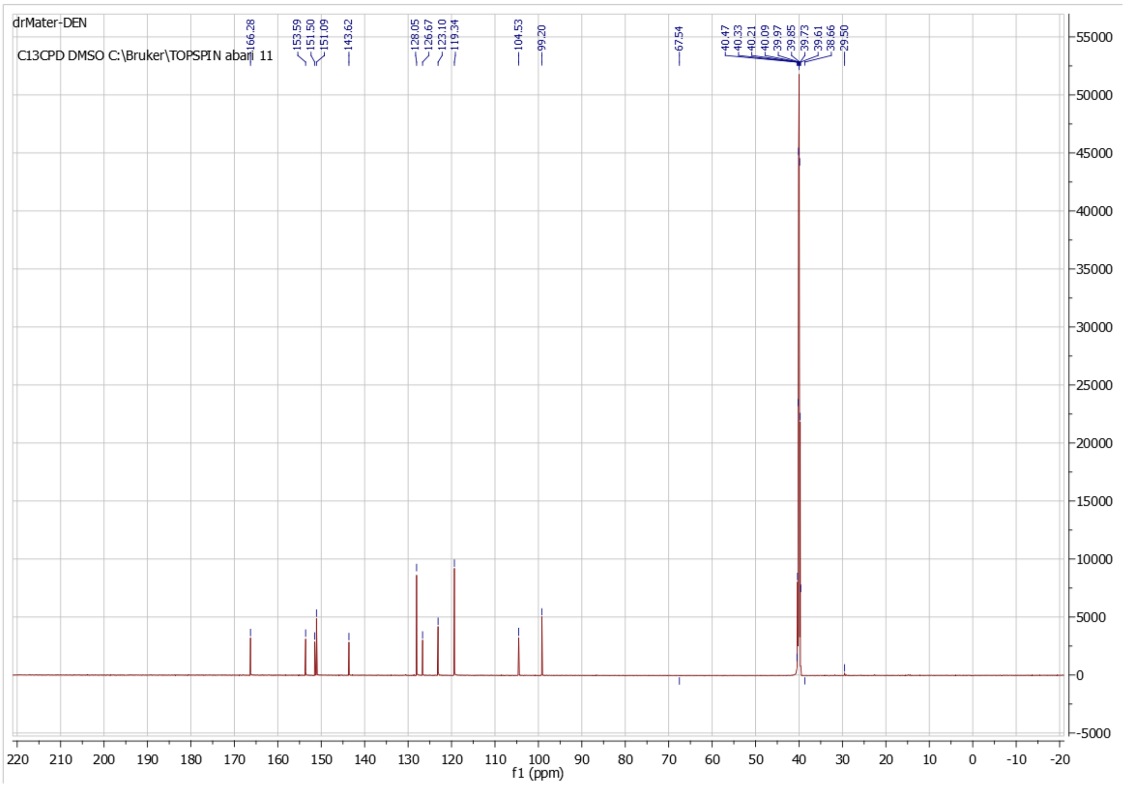

LCMS negative

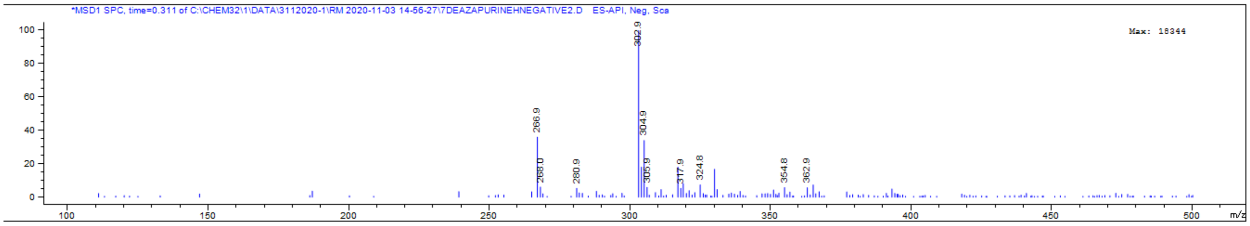

LCMS Positive

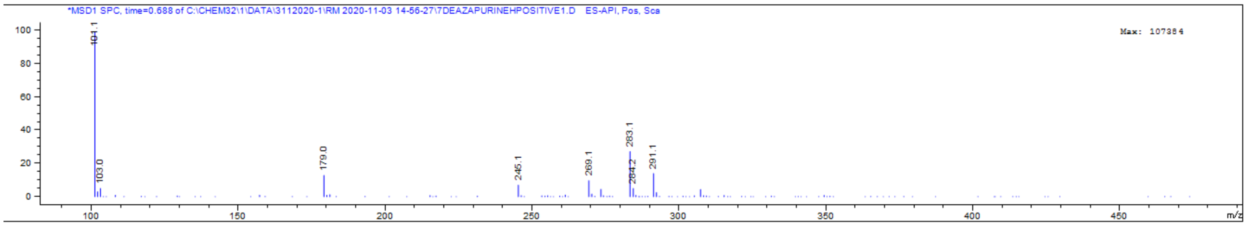

2. ADMET profile

Table 1. Physicochemical properties and ADMET profiles of isatin-deazapurine hybrid compounds **1-5 and 9-12**, calculated by pkCSM.

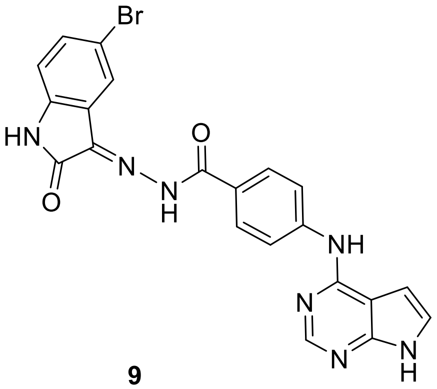

9

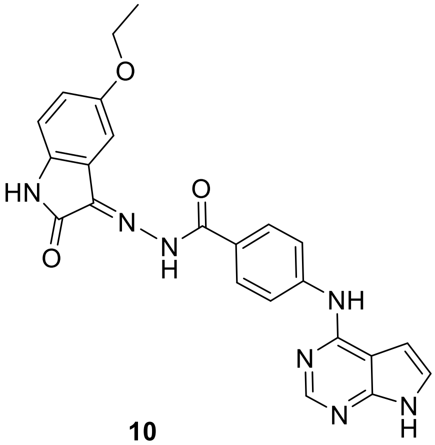

10

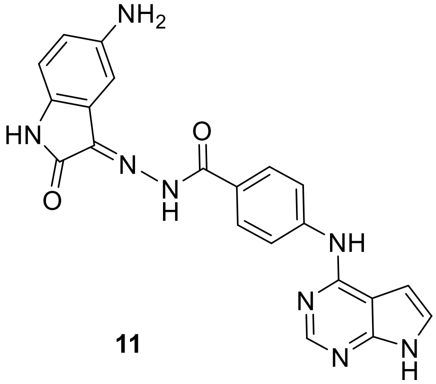

11

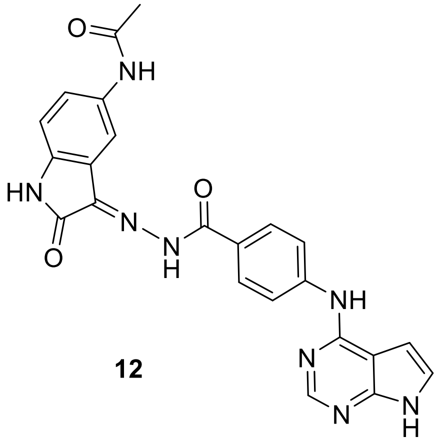

12

Parameters / Compound	1	2	3	4	5	9	10	11	12	Sunitinib
**Molecular properties**										
Molecular weight	397.398	431.843	415.388	411.425	427.424	476.294	441.451	412.413	454.45	398.482
LogP	2.7877	3.4411	2.9268	3.0961	2.7963	3.5502	3.1864	2.3699	2.7461	3.33494
H-acceptor	6	6	6	6	7	6	7	7	7	3
H-donors	4	4	4	4	4	4	4	5	5	3
Surface area	169.803	180.106	173.968	176.168	181.281	183.670	187.646	175.143	192.244	169.730
**Absorption**										
Water solubility	-3.118	-3.122	-3.117	-3.127	-3.116	-3.123	**-3.119**	-2.922	-2.942	-4.338
Intestinal absorption (human)	79.999	81.175	78.38	80.467	76.475	80.911	77.244	72.856	72.434	94.534
Skin permeability	-2.735	-2.735	-2.735	-2.735	-2.735	-2.735	-2.735	-2.735	-2.735	-2.79
**Distribution**										
BBB permeability	-1.325	-1.507	-1.486	-1.354	-1.476	-1.528	-1.517	-1.558	-1.734	-1.122
CNS permeability	-2.474	-2.367	-2.541	-2.407	-2.667	-2.344	-2.679	-2.626	-2.721	-2.601
**Metabolism**										

CYP1A2 inhibitor	YES	YES	YES	YES	YES	YES	YES	YES	YES	NO
CYP2C19 inhibitor	YES	YES	YES	YES	YES	YES	YES	YES	YES	NO
CYP2C9 inhibitor	YES	YES	YES	YES	YES	YES	YES	YES	YES	NO
CYP2D6 inhibitor	NO	NO	NO	NO	NO	NO	NO	NO	NO	YES
CYP3A4 inhibitor	YES	YES	YES	YES	YES	YES	YES	YES	YES	YES
**Excretion**										
Total clearance	-0.022	-0.281	0.08	-0.018	0.186	-0.303	0.236	0.012	0.07	1.055
Renal OCT2 substrate	NO	NO	NO	NO	NO	NO	NO	NO	NO	NO
**Toxicity**										
Max. tolerated dose (human)	0.587	0.626	0.621	0.616	0.635	0.626	0.629	0.673	0.687	0.223
Oral rate acute toxicity (LD50)	2.837	2.528	2.773	2.854	2.776	2.851	2.794	2.373	2.682	2.387
Oral rate chronic toxicity (LOAEL)	2.651	2.528	2.363	2.481	2.314	2.511	2.314	2.628	2.567	1.662
Hepatotoxicity	YES	YES	YES	YES	YES	YES	YES	YES	YES	YES
Skin sensitization	NO	NO	NO	NO	NO	NO	NO	NO	NO	NO

Physicochemical properties and DMET profiles of 1, calculated by pkCSM: https://biosig.lab.uq.edu.au/pkcsm/prediction_single/adme_1683988765.63

Physicochemical properties and DMET profiles of 2, calculated by pkCSM: https://biosig.lab.uq.edu.au/pkcsm/prediction_single/adme_1683989159.67

Physicochemical properties and DMET profiles of 3, calculated by pkCSM: https://biosig.lab.uq.edu.au/pkcsm/prediction_single/adme_1683989502.25

Physicochemical properties and DMET profiles of 4, calculated by pkCSM: https://biosig.lab.uq.edu.au/pkcsm/prediction_single/adme_1683989897.49

Physicochemical properties and DMET profiles of 5, calculated by pkCSM: https://biosig.lab.uq.edu.au/pkcsm/prediction_single/adme_1683990209.98

Physicochemical properties and DMET profiles of 9, calculated by pkCSM: pkCSM (uq.edu.au)

Physicochemical properties and DMET profiles of 10, calculated by pkCSM: pkCSM (uq.edu.au)

Physicochemical properties and DMET profiles of 11, calculated by pkCSM: pkCSM (uq.edu.au)

Physicochemical properties and DMET profiles of 112, calculated by pkCSM: pkCSM (uq.edu.au)

3. Docking study

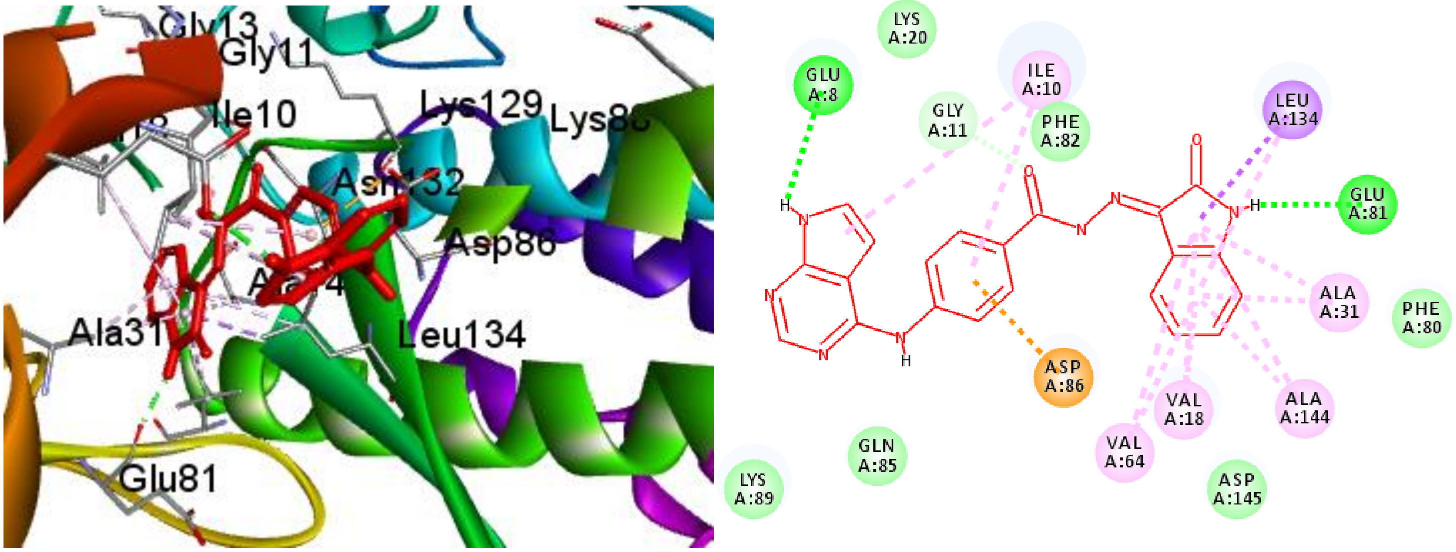

figure S1. 3D and 2D interactions of compound **1** in the active site of CDK2. Green dotted lines represent hydrogen bonds.

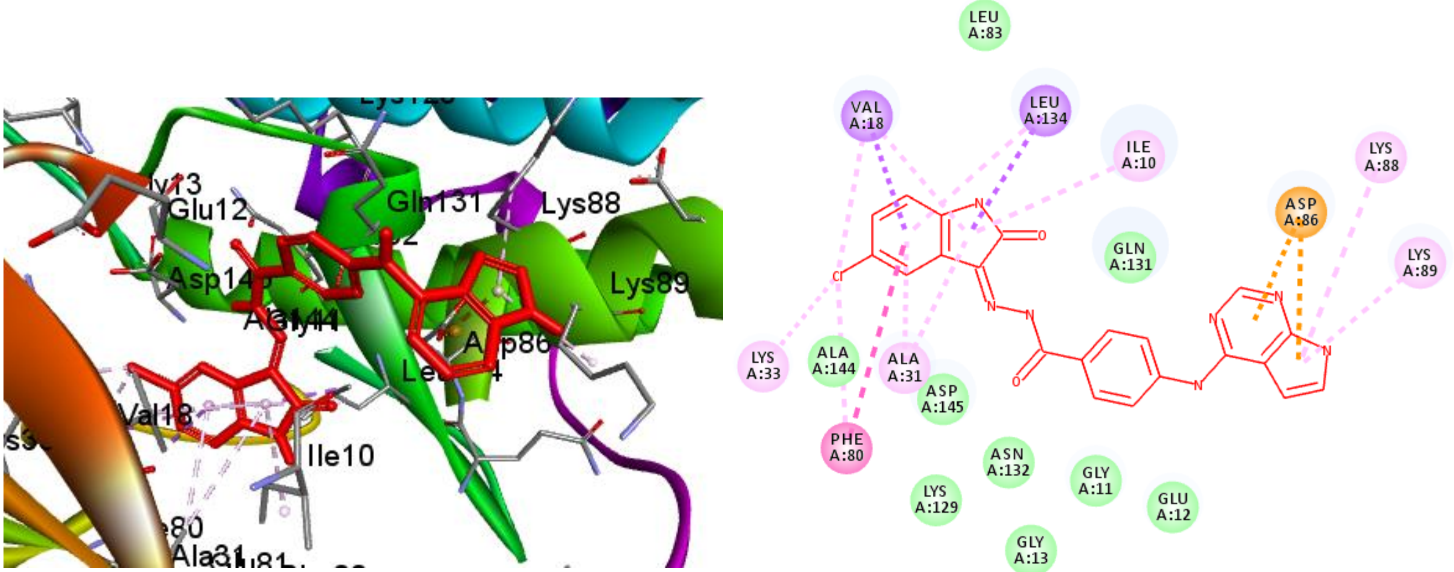

Figure S2. 3D and 2D interactions of compound **2** in the active site of CDK2. Green dotted lines represent hydrogen bonds.

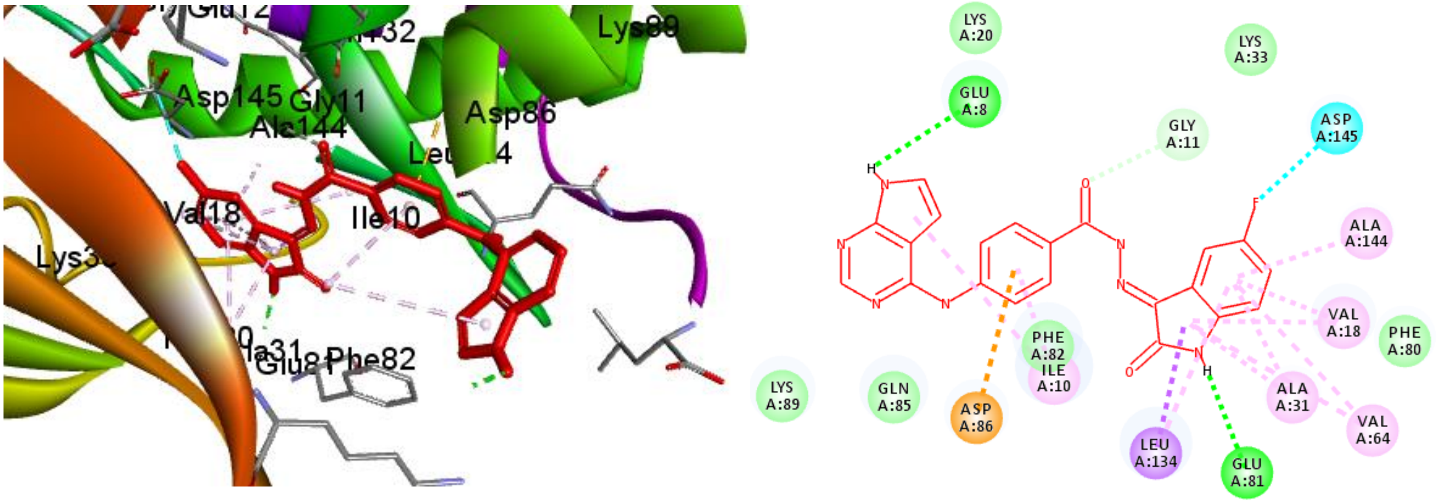

Figure S3. 3D and 2D interactions of compound **3** in the active site of CDK2. Green dotted lines represent hydrogen bonds.

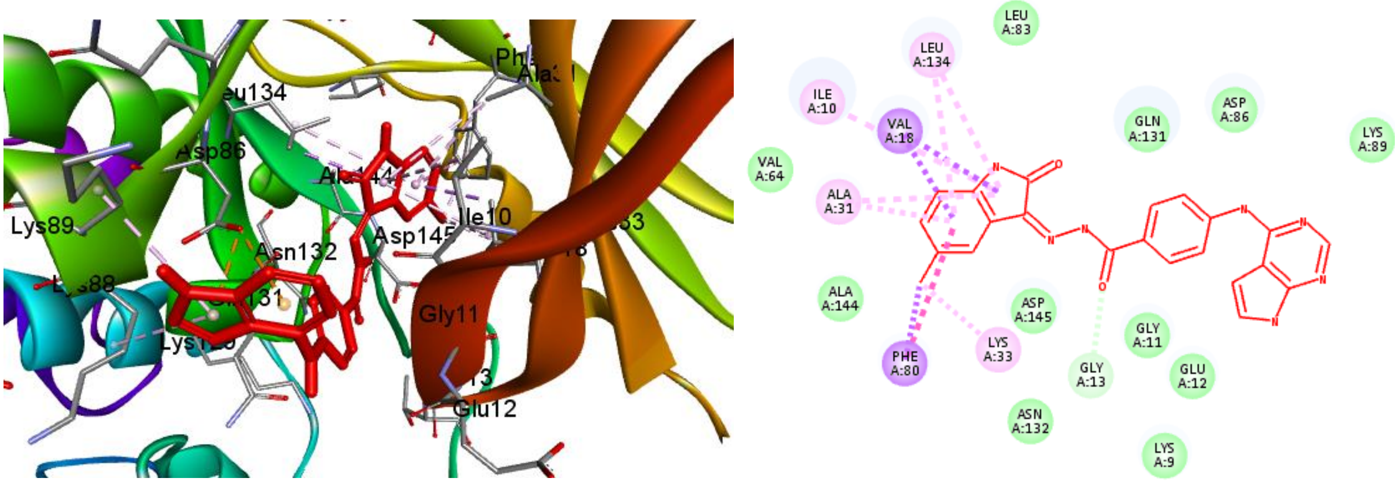

Figure S4. 3D and 2D interactions of compound **4** in the active site of CDK2. Green dotted lines represent hydrogen bonds.
